# Effect of Plant Extracts on the Characteristics of Silver Nanoparticles for Topical Application

**DOI:** 10.3390/pharmaceutics12121244

**Published:** 2020-12-21

**Authors:** Ioanna K. Siakavella, Fotini Lamari, Dimitrios Papoulis, Malvina Orkoula, Patroula Gkolfi, Michail Lykouras, Konstantinos Avgoustakis, Sophia Hatziantoniou

**Affiliations:** 1 Laboratory of Pharmaceutical Technology, Department of Pharmacy, School of Health Sciences, University of Patras, 26504 Patras, Greece; ioanna.siakav@gmail.com (I.K.S.); avgoust@upatras.gr (K.A.); 2 Laboratory of Pharmacognosy & Chemistry of Natural Products Department of Pharmacy, School of Health Sciences, University of Patras, 26504 Patras, Greece; flam@upatras.gr; 3 Department of Geology, University of Patras, 26504 Patras, Greece; papoulis@upatras.gr; 4 Laboratory of Instrumental Pharmaceutical Analysis, Department of Pharmacy, School of Health Sciences, University of Patras, 26504 Patras, Greece; malbie@upatras.gr (M.O.); michalislyk@gmail.com (M.L.); 5 Department of Chemistry, University of Patras, 26504 Patras, Greece; patroula.gkolfi@gmail.com

**Keywords:** silver nanoparticles, AgNP, green chemistry, plant extracts, topical application, skin permeation

## Abstract

Silver nanoparticles (AgNPs) were synthesized using hydroalcoholic extracts of dittany (*Origanum dictamnus*), sage (*Salvia officinalis*), sea buckthorn (*Elaeagnus rhamnoides*, syn. *Hippophae rhamnoides*), and calendula (*Calendula officinalis*) as reducing agents. AgNPs synthesized using NaBH_4_ and citric acid were used as control. The impact of the origin of the extract and preparation conditions (light, temperature, reaction time) on the properties of the synthesized AgNPs was investigated. The structure, morphology, composition, physicochemical characteristics, and colloidal stability were characterized using dynamic laser scattering (DLS), ultraviolet-visible spectrophotometry (UV–/Vis), XRD, X-ray fluorescence (XRF), TEM, and FTΙR. The reduction of total phenolic and flavonoid content of the extracts after the reaction of AgNPs synthesis was also determined. Low IC50 values for all types of AgNPs revealed good antioxidant activity, attributable to the phenolic and flavonoid content of their surface. The results suggest that plant extract selection is important to the green synthesis of AgNPs because it affects the kinetics of their synthesis as well as their morphology, physicochemical characteristics, and colloidal stability. In vitro permeation studies on porcine skin revealed that AgNPs remained at the upper layers of stratum corneum and did not penetrate the skin barrier after 4 h of cutaneous application suggesting the safety of their application on intact skin for a relatively short time.

## 1. Introduction

Metallic nanoparticles have recently gained a lot of interest due to their unique physical and chemical properties [1]. These properties are due to the huge surface/volume ratio and depend on the size and shape of the nanoparticles [2].

Silver nanoparticles (AgNPs) are among the most interesting nanotechnology products as they possess unique properties such as conductivity and good chemical stability, as well as antimicrobial, antiviral, anti-inflammatory, antioxidant, and anticancer activity. Especially in wound healing, their use as antibiotics’ substitution is widely studied [3]. All the above has triggered the broad use of the AgNPs in pharmaceutical and cosmetic products, the food industry, etc. They are used in a wide variety of products such as creams, wound healing patches, and sprays for topical application [2]. They have also been proposed as antimicrobial agents in cosmetics [4,5] and textile coatings [6].

Numerous methods have been reported for the synthesis of AgNPs. The method known as chemical reduction uses reducing agents such as NaBH_4_ or citric acid for the reduction of AgNO_3_ and polymers (PEG) for the stabilization of the particles [7]. Other extensively used methods are thermal decomposition [8], microwave-assisted process [9], and electrochemical reduction [10]. These methods are costly and have a considerable burden on the environment, especially due to the chemicals that are used during the synthesis. This has led researchers towards the pursuit of more environmentally friendly and low-cost methods such as the use of microorganisms, plants, and plant extracts for the green synthesis of AgNPs [11,12].

The synthesis of AgNPs assisted by plant extracts is considered the best biologically based method. The ability of plant extracts to reduce metallic ions has been known since the early 1900s but only recently the mechanism was elucidated. It seems that the reduction of Ag is caused by metabolites such as polyphenols, alkaloids, amino acids, and carbohydrates that exist in the plant extracts [13]. They not only participate in the reduction but also act as capping and stabilizing agents preventing the aggregation of the nanoparticles. The plant extract can affect the synthesis in many ways depending on its chemical composition. 

Although there is extensive literature concerning the preparation of NPs using plant extracts, the reported preparation conditions differ significantly while to our knowledge a study on the impact of the origin of the extract and the conditions of preparation on the properties of the prepared AgNPs is lacking.

The aim of this work was to prepare AgNPs using green chemistry and hydroglycolic extracts of medicinal plants and to investigate the impact of each extract on the properties of the prepared nanoparticles. Specifically, the hydroglycolic extracts of sea buckthorn (*Elaeagnus rhamnoides* (L.) A.Nelson, syn. *Hippophae rhamnoides* L.), dittany (*Origanum dictamnus* L.), sage (*Salvia officinalis* L.), and calendula (*Calendula officinalis* L.) were applied as reducing agents. These extracts are widely used by the cosmetic industry in Greece due to their unique beneficial properties but have never been investigated as reducing agents for the synthesis of AgNPs. As control, AgNPs were synthesized using NaBH_4_ as reducing agent. The synthesized AgNPs were characterized using dynamic laser scattering (DLS), electrophoretic laser scattering (ELS), ultraviolet-visible spectrophotometry (UV-Vis), X-ray diffraction (XRD), total reflection X-ray fluorescence (TXRF), Fourier transform infrared spectroscopy (FTΙR), and transmission electron microscopy (TEM). The radical scavenging potential of the AgNPs as well as the total phenolic (TPC) and flavonoid (TFC) content of the extracts before and after the synthesis reaction were determined. Finally, skin permeation studies were performed on porcine ears skin using vertical diffusion cell (Franz cells) and tape-stripping techniques.

## 2. Materials and Methods 

All the chemicals used were of analytic grade. Silver nitrate (Fisher Scientific, Leicester, UK), 2,2-diphenyl-1-picrylhydrazyl (DPPH) (Sigma-Aldrich, Darmstadt, Germany), water for injection (WFI) (Demo S.A., Pharmaceutical Industry, Kryoneri, Attica, Greece), water ultra-pure (Chem-Lab NV, Zedelgem, Belgium), methanol (Fisher Scientific UK (Leicester, UK)), Folin-Ciocalteu’s reagent (PanReac AppliChem, Darmstadt, Germany), gallic acid (Fluka Chemika Chemie GmbH, Buchs, Switzerland), Na_2_CO_3_ (Chem-Lab NV, Zedelgem, Belgium), quercetin dihydrate (Extrasynthese, Genay, France), potassium acetate (PanReac AppliChem, Darmstadt, Germany), AlCl_3_ (Sigma-Aldrich, Darmstadt, Germany), ascorbic acid (Sigma-Aldrich, Darmstadt, Germany), NaBH_4_ (Fluka Chemie GmbH, Buchs, Switzerland), citric acid (Sigma-Aldrich, Darmstadt, Germany), and phosphate-buffered saline (PBS) (Sigma-Aldrich, Darmstadt, Germany) were purchased. Extracts were commercially available cosmetic grade ingredients and were donated by Access Naturals, Athens, Greece.

### 2.1. Synthesis of AgNPs Using Plant Extracts

The synthesis of AgNPs was achieved using an AgNO_3_ solution (10 mM) and a plant extract in a ratio of 3:2 by volume. To determine the best reaction conditions for each extract, the impact of light, heating, and time was assessed. The reaction mixtures were left under stirring for 24 h on a thermal magnetic stirrer with or without visible light or heating (50–60 °C). The time of the reaction completion was determined by assessing the presence of AgNPs at predetermined time intervals by spectrophotometry. The solution was then centrifuged at 13,000× *g* rpm for 15 min at 15 °C. The sediment was collected and dispersed in WFI. The procedure was repeated twice, and the combined dispersions were stored at 4 °C for further analyses. 

### 2.2. Synthesis of AgNPs Using a Conventional Reducing Agent

The synthesis of AgNPs using a conventional reducing reagent was made according to the protocols reported by Banne et al. (2017) and Pinto et al. (2010) with minor modifications [14,15]. Three aqueous solutions were used (10 mM AgNO_3_, 20 mM NaBH_4_, and 0.6 mM sodium citrate) in volume ratio 1.25:1:1. Initially, NaBH_4_ and sodium citrate solutions were mixed in an ice bath placed under stirring (300 rpm, 30 min). The silver nitrate solution was then added dropwise. The reaction solution was left without stirring for 24 h. The process of purifying and recovering AgNPs was the same as mentioned above (2.1).

### 2.3. Monitoring of AgNPs Synthesis by Spectrophotometry 

The synthesis of the AgNPs was monitored by spectrophotometry (UV-1800 UV–Vis Spectrophotometer, SHIMADZU, Kyoto, Japan). The presence of an absorption peak at 420–480 nm, which is due to the collective oscillation of the surface electrons surface plasmon resonance (SPR) of metallic silver, confirms the synthesis of AgNPs [11,14,15].

### 2.4. Physicochemical Characterization and Stability Study of AgNPs

The average hydrodynamic diameter (mean size) and ζ-potential of AgNPs was monitored by DLS or ELS, respectively (Malvern Instruments Ltd., Malvern, UK) after their dispersion in WFI. The colloidal stability of the AgNPs was assessed by storing the dispersions at 4 °C and measuring their size and zeta potential at predetermined time intervals for a period of 120 days. 

### 2.5. Morphology of AgNPs

The morphology of AgNPs was investigated by TEM (JEOL: JEM-2100, Tokyo, Japan). The size of AgNPs from the electron microscopy images was analyzed using Fiji program (ImageJ 1.50d, an open source software, National Institutes of Health, Bethesda, MD, USA) [16] and graphs of the AgNPs’ size distributions were constructed. Additionally, the selected area (electron) diffraction (SAED) patterns of AgNPs were obtained revealing the distance between the refraction centers and proving the crystalline structure of AgNPs.

### 2.6. XRD, tXRF and FTΙR Study of AgNPs

The crystalline nature of the nanoparticles was determined by XRD (Bruker D8 Advance Diffractometer, Berlin, Germany). 

Elemental analysis of AgNPs was performed employing total reflection X-ray fluorescence spectroscopy (TXRF) using a benchtop total-reflection X-Ray fluorescence spectrometer (S2 PICOFOXTM, Bruker Nano GmbH, Berlin, Germany) equipped with a molybdenum (Mo) target X-ray tube operating at 30 W (50 KV, 0.6 mA).

Gallium was chosen as the internal standard. The spectra were processed using the S2 PicofoxTM software (Bruker Nano GmbH, Berlin, Germany).

To assess the presence of functional groups in the surface of AgNPs, FTIR spectra (4000–400 cm^−1^) were recorded using a Perkin–Elmer (Perkin-Elmer, Watham, MA, USA) 16 PC spectrometer with samples prepared as KBr pellets. To overcome the difficulty of freeze-drying the extracts due to the presence of glycerin, they were allowed to be absorbed on active carbon. The FTΙR pattern of active carbon alone or loaded with each extract was also obtained as control.

### 2.7. Determination of Total Phenolic Content (TPC)

The determination of TPC was performed according to the Folin and Ciocalteu (1927) and Ainsworth and Gillepsie (2007) protocols [17,18]. Various concentrations of the initial extracts (33.3, 20.0, 16.67, 11.1, and 9.09 mg dry plant/mL) and the supernatants (13.3, 8.0, and 5.71 mg dry plant/mL) derived from the reactions after the purification of the AgNPs were tested. Twenty (20) μL of the samples were mixed with 40 μL Folin-Ciocalteu reagent and 160 μL Na_2_CO_3_ (7.5% *w/v*). After incubation of the reaction mixtures for 60 min in 25 ℃ in the dark, the absorbance at 620 nm was measured using an absorbance microplate reader (SunriseTM TECAN Trading AG, Männedorf, Switzerland). All the reactions were carried out in a 96-well plate and they were repeated 3 times. Gallic acid was used as a standard and the data were expressed as milligrams of gallic acid equivalents.

### 2.8. Determination of Total Flavonoid Content (TFC)

The determination of TFC was performed according to the Woisky and Salatino (1998) protocol [19]. Various concentrations of the initial extracts (33.3, 20.0, 16.67, 11.1, and 9.09 mg dry plant extract/mL) and the supernatants (13.3, 8.0, and 5.71 mg dry plant/mL) derived from the reactions after the purification of the AgNPs were tested. Sixteen (16) μL of the samples were mixed with 5 μL CH_3_COOK 1 M, 5 μL AlCl3 10% *w/v*), 40 μL of ethanol 95%, and 75 μL ultrapure water. After incubation of the reaction mixtures for 45 min at RT and in absence of light, the absorbance at 405 nm was measured using an absorbance microplate reader (SunriseTM TECAN Trading AG, Männedorf, Switzerland). All the reactions were repeated 3 times. Quercetin was used as a standard and the data were expressed as milligrams of quercetin equivalents.

### 2.9. Antioxidant Activity

Free radical scavenging activity was determined using the DPPH method as described by Brand-Williams (1995) in association with the Kim and Lee (2002) protocol [20,21]. In 195 μL of 0.1 mM DPPH methanolic solution, 5 μL of AgNPs at various concentrations (500, 250, 125, 100, 50, and 25 μg Ag/mL) were added. The reaction was performed at 25 °C for 30 min in the dark. All the reactions took place on a 96-well plate in triplicate and the absorbance at 540 nm was measured using an absorbance microplate reader (SunriseTM TECAN Trading AG, Zürich, Switzerland). The obtained data were analyzed using Microsoft Office Excel 2007 (Redmond, WA, USA). The IC50 values were defined as the minimum quantity of antioxidant required to scavenge 50% of the DPPH free radicals. Ascorbic acid in concentrations ranging from 0.007 to 0.25 mg/mL was used as positive control. The DPPH free radical scavenging activity was calculated according to Equation (1).

Ι% = 100 − (Abs(control) − Abs (sample))/Abs(control) × 100,(1)

where Abs (sample) is the absorbance of the reacted mixture of DPPH with the extract sample and Abs (control) is the absorbance of the DPPH solution. 

### 2.10. Skin Penetration Studies 

#### 2.10.1. Vertical Diffusion Cells (Franz Cells)

Porcine ears were obtained from a local abattoir (Corinth, Greece) immediately post sacrifice. The skin was excised from the underling cartilage, cut in circles of 9 mm diameter and fitted on Franz cells. In the recipient cell, 5 mL of PBS (pH 7.4) was added. The Franz cells were mounted onto magnetic stirrers (400 rpm) d at 37 °C. After, the system was mounted and 200 μL of an aqueous dispersion containing 10 μg AgNPs was added onto the skin surface. The applied quantity corresponds to 15.62 μg AgNPs/cm^2^. Samples of PBS were collected after 4 h and analyzed by TXRF for the potential presence of Ag. The presence of other elements was also tested.

#### 2.10.2. Tape Stripping

After the skin diffusion study, the skin disks were demounted from the Franz cells and the stratum corneum (SC) was removed by tape stripping using adhesive tape (Tesafilm transparent Universal, code: 57341-00008, Tesa SE Norderstedt, Norderstedt, Germany). In total, 15 tape strippings were obtained and were placed in a test tube with 5 mL methanol and left overnight. The extracts were then analyzed using TXRF. The mean weight of the SC that was removed was correlated to 30% of total SC.

### 2.11. Statistical Analysis

The results are reported as mean values ± standard deviation (SD) of measurements executed in triplicate, while the statistical significance of differences was calculated using Student’s *t*-test (Microsoft Office Excel 2007, Redmond, WA, USA). 

## 3. Results

### 3.1. Synthesis of the AgNPs Monitored by Spectrophotometry

The synthesis of AgNPs was detected using UV-Vis spectrometry. All the AgNPs derived from extracts showed an absorbance peak at 420–480 nm (Supplementary Materials Figure S1a–d). The NaBH_4_ AgNPs showed an absorbance peak at 399 nm (Supplementary Materials Figure S1e). The redshift observed in the case of AgNPs that are synthesized using extracts may be explained by the difference in their size, shape, and agglomeration status [22]. Our results are in accordance with results obtained previously with different extracts [15,23,24,25].

### 3.2. Synthesis of AgNPs Using Plant Extracts

#### 3.2.1. Effect of Light on the Synthesis of AgNPs

The optimal conditions for the synthesis of AgNPs from each extract were investigated. The first condition tested was the presence of light. After the reaction in the dark at RT, the presence of AgNPs in the mixtures was investigated using spectrophotometry and the obtained absorption spectra revealed the absence of the characteristic peak at 420–480 nm (Supplementary Materials Table S1 and Figure S2I). These results indicated that in the absence of light, AgNPs were not synthesized. On the contrary, when the reaction was allowed to take place under light, the synthesis of AgNPs was confirmed by their color change (Supplementary Materials Figure S3) and the presence of their characteristic peak. Under light at RT for 24 h, all the extracts could reduce silver to AgNPs (Supplementary Materials Figure S2II), indicating that light is critical in order to produce AgNPs. Since all reactions require some form of energy in order to proceed, we may assume that light offers the needed amount of energy. The importance of light exposure is also mentioned in Bhaduri et al. (2013) [26].

#### 3.2.2. Effect of Heating on the Synthesis of AgNPs

The next step was to test the effect of heating at 50–60 °C on the reaction. The reaction mixtures of the extracts were heated under light for a short time (1–2 h) and the formation of NPs was monitored spectrophotometrically. It was noticed that AgNPs were synthesized after 1 h of heating of dittany, sage, and sea buckthorn extracts. On the contrary, while calendula AgNPs were synthesized under light at RT for 24 h (Supplementary Materials Table S1 and Figure S2d), when heating was applied in this reaction mixture under light, AgNPs were not detectable even if the heating time was extended to 2 h (Supplementary Materials Figure S4). Based on these results, it was concluded that the right combination of light and heating for each extract is essential for the successful synthesis of AgNPs.

#### 3.2.3. Kinetics of the Synthesis of AgNPs

In order to determine the time needed to produce the maximum amount of AgNPs from each extract, a kinetic experiment was conducted. The reactions took place under light for 24 h, with or without heating. At predetermined time points (1, 3, 5, and 24 h), a sample was withdrawn and the presence of AgNPs was examined spectrophotometrically (Supplementary Materials Figures S5–S8). The time point beyond which no further increase of the absorption was noted was set as the time of completion of the reaction (Supplementary Materials Figure S9). The color change over time of the reaction mixtures of each extract due to the formation of AgNPs is demonstrated in Supplementary Materials Figure S3. 

Based on these results, the optimal conditions for each extract were determined. The AgNPs generated from dittany were optimally synthesized at 24 h without heating under light (Supplementary Materials Figure S5). Despite the prompt synthesis of AgNPs when heat was applied, we considered that a green approach without the need of extra energy (heating) was preferable. The same reaction conditions were chosen for the extracts of sage and calendula for the same reason (Supplementary Materials Figures S6 and S8 respectively). Only in the synthesis with the extract of sea buckthorn it was decided to apply heat for 24 h under light because the reaction was not complete after 24 h without heating (Supplementary Materials Figure S7). 

### 3.3. Physicochemical Characterization of AgNPs

#### 3.3.1. Size and ζ-Potential

The size and ζ-potential of the AgNPs was monitored by DLS or ELS, respectively. The mean size and ζ-potential of AgNPs immediately after their synthesis are summarized in Table 1. The size of AgNPs ranged from 76.90 ± 0.14 nm (NaBH_4_ AgNPs) to 364.6 ± 13.09 nm (calendula AgNPs). The Polydispersity Index (PDI) ranged from 0.269 ± 0.006 (sea buckthorn) to 0.441 ± 0.008 (NaBH_4_). With the plant extracts, the smallest (*p* < 0.05) AgNPs were obtained with the sage extract while calendula AgNPs were the largest (*p* < 0.05) AgNPs. The ζ-potential’s absolute value was high for all AgNPs (−26.87 mV ± 0.29 mV for NaBH_4_ to −31.5 mV ± 0.72 mV for calendula), indicating good colloidal stability. Because AgNPs were dispersed in water, the negative charge of ζ-potential may be attributed to functional groups of biomolecules attached on the surfaces of AgNPs, such as -OH from flavones or -COO from citric acid in case of NaBH_4_ AgNPs [27].

#### 3.3.2. Stability Studies

Both size and ζ-potential for all AgNPs were unaltered (*p* > 0.05), indicating good colloidal stability when stored at 4 °C for at least 120 days. While the mean size of NaBH_4_ and calendula AgNPs remained practically unaltered, a significant increase in PDI may be an indication of low degree of aggregation and destabilization after 90 d and 120 d respectively (Supplementary Materials Table S2).

#### 3.3.3. Morphology of AgNPs

**Figure 1:**
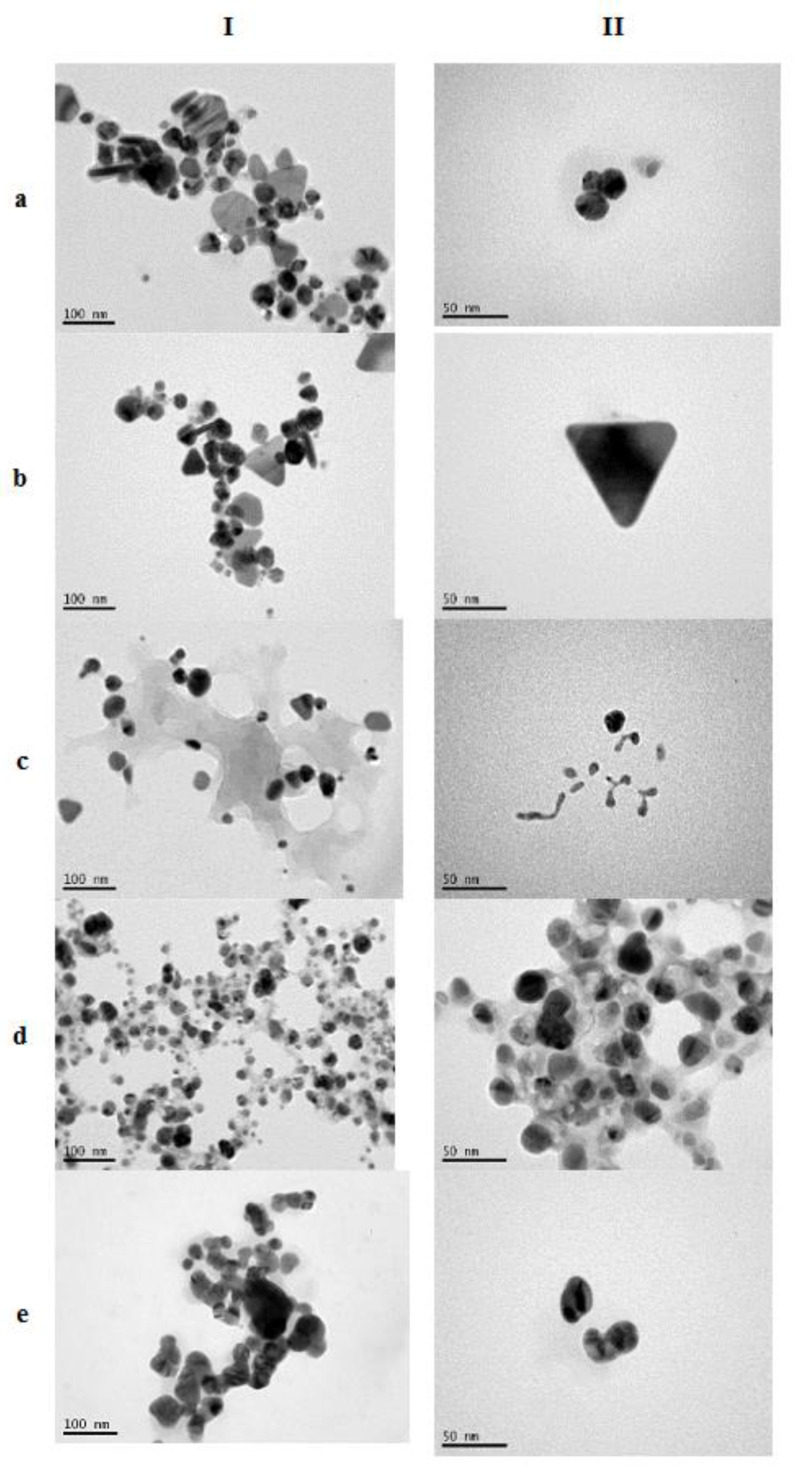
TEM images of (**a**) dittany, (**b**) sea buckthorn, (**c**) sage, (**d**) calendula, and (**e**) NaBH_4_ AgNPs observed under × 200,000 (**I**) or × 500,000 (**II**) magnitude. Bar represents 100 nm (**I**) and 50 nm (**II**).

The TEM images of AgNPs (Figure 1) revealed a wide diversity in their morphology that may be attributed to the differences of the extracts in the composition of the extracts. Dittany and sage AgNPs have similar morphology consisting of mixed shapes of particles. Spherical, triangular, polygonal but also some rod-shaped particles can be observed. Based on the literature, these shapes are characteristic of AgNPs that are synthesized using either NaBH_4_ or plant extracts [22,28]. Sea buckthorn AgNPs have a completely different morphology consisting mainly of some spherical but mostly of rod-shaped particles while calendula and NaBH_4_ AgNPs are spherical. The nanoparticles images obtained with calendula extract are similar to the ones synthesized previously using calendula extract [29].

**Figure 2:**
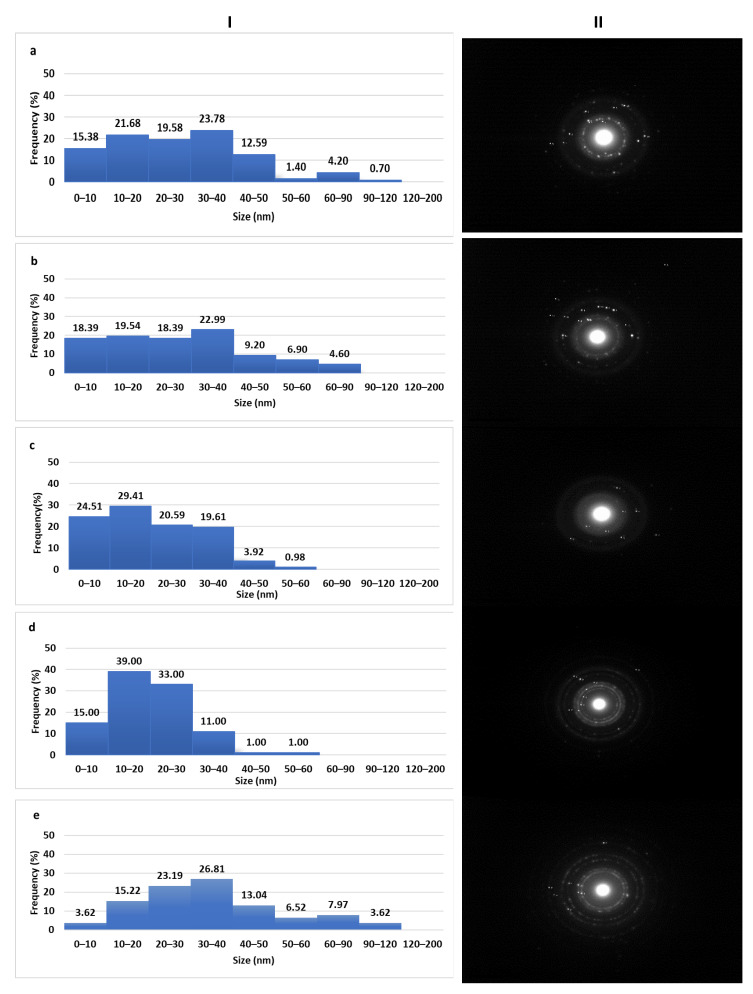
Size distribution obtained by TEM image analysis (**I**) and selected area (electron) diffraction (SAED) patterns (**II**) of dittany (**a**), sage (**b**), sea buckthorn (**c**), calendula (**d**), and NaBH_4_ AgNPs (**e**).

The sizes of AgNPs that were calculated by analysis of the TEM images were quite small and in most cases below 100 nm (Figure 2). These results differ from those obtained by DLS particularly in the case of sea buckthorn and calendula AgNPs. These divergences, however, can be explained because DLS measures the hydrodynamic diameter of the particles. Moreover, the nanoparticles are very close to each other forming aggregates, which in the DLS may be detected as larger particles. 

Additionally, the presence of a thick capping layer resembling a transparent halo around the AgNPs was observed in the TEM images. This halo seemed to surround them and may be attributed to the organic compounds bound on the nanoparticles’ surfaces. The constituents of this capping layer may derive from plant extracts or, in the case of NaBH_4_, AgNPs from sodium citrate. Its presence is connected to the long-term colloidal stability of the AgNPs [27].

#### 3.3.4. Crystallinity of AgNPs

Based on the obtained SAED patterns (Figure 2), the d-spacing between the lattices of AgNPs was calculated (Supplementary Materials Table S3). The diffraction ring patterns and d-spacing values were correlated to the (111), (200), (220), and (311) lattice planes which is typical of the polycrystalline structure and confirms the synthesis of AgNPs and suggest face-centered cubic (FCC) structure. The fact that the lattice planes (200) and (311) were not detected in SAED patterns for calendula and sea buckthorn AgNPs respectively, may be attributed to the slow rate of AgNPs synthesis in both cases [27]. The presence of AgCl that was detected in the sage, sea buckthorn, and calendula AgNPs may be explained by the existence of small amounts of Cl- in the extracts.

**Figure 3:**
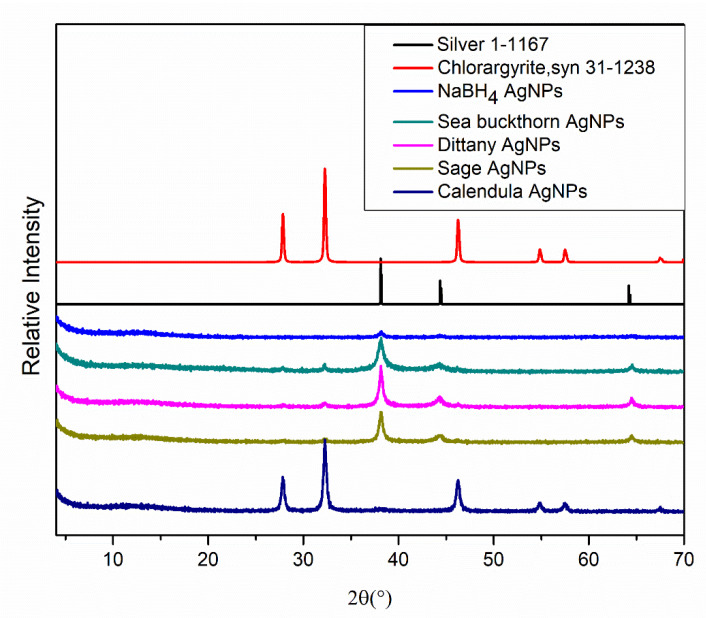
XRD spectra of Ag, AgCl, NaBH_4_, sea buckthorn, dittany, sage, and calendula AgNPs.

XRD was used to determine the crystalline nature of the AgNPs. The main peaks at (2θ) 38.13, 44.32, and 64.58 that correspond to (111), (200), and (220) planes respectively were present on the XRD patterns of dittany, sage, sea buckthorn, and NaBH_4_ AgNPs indicating similarity of their crystalline structure (Figure 3). On the contrary, the XRD pattern of calendula AgNPs indicates the presence of AgCl due to the characteristic peaks at (2θ) 46.22 and 54.91 that correspond to (220) and (222) planes of AgCl crystals. Based on this observation, it was concluded that when calendula was used, the main constituent of the sample was AgCl and only a low amount of AgNPs were synthesized. The XRD pattern of the calendula AgNPs is also confirmed [29]. These results were in accordance with the ones obtained from the SAED patterns study.

#### 3.3.5. Chemical Composition of AgNPs

Elemental analysis was performed for all AgNPs samples. Chlorine (Cl), potassium (K), and calcium (Ca) elements were the most abundant. Phosphorous (P) was only detected in PBS dispersed AgNPs. Sodium (Na) was impossible to measure due to its small atomic number (concentration lower to the detection limit of the element).

Silver (Ag) presence was verified in all samples at concentrations summarized in Table 2. Ag concentration ranged from 315.6 ± 37.3 ppm (NaBH_4_ AgNPs) to 821.7 ± 171.6 ppm and 825.5 ± 84.6 ppm for dittany and sage AgNPs, respectively. Although the calendula AgNPs had high Ag concentration (863.1 ± 33.1 ppm), their concentration of Cl was also higher compared to the other AgNPs. In order to detect the origin of Cl in the AgNPs, TXRF analysis was performed to the extracts (Table 2). The results revealed that Cl- concentration in the calendula was significantly higher than the other extracts. This result confirmed the participation of Ag mainly as AgCl and not in its metallic form, as revealed by XRD and SAED pattern analysis, and may explain the difficulty of the synthesis of AgNPs from the calendula extract.

**Figure 4:**
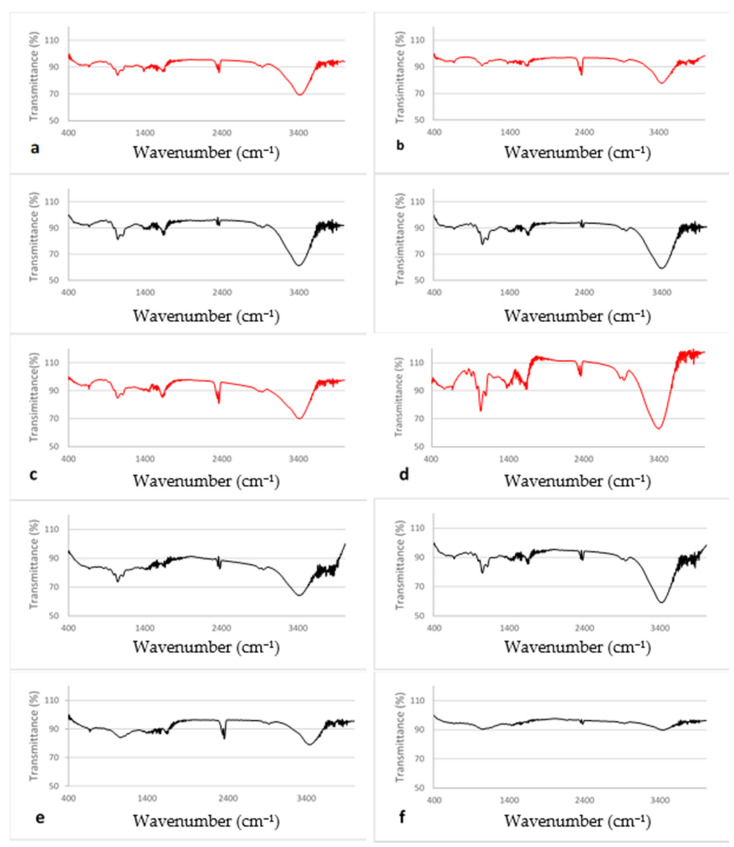
FTΙR spectra of (**a**–**d**) extract AgNPs (-) and extract absorbed on active carbon (-) for (**a**) dittany, (**b**) sage, (**c**) sea buckthorn, (**d**) calendula, (**e**) NaBH_4_ AgNPs, and (**f**) active carbon.

The functional groups that participated in the reduction of Ag ions or in the stabilization of the synthesized AgNPs were studied using FTΙR spectroscopy. The spectrum of each extract absorbed on active carbon and the relevant AgNPs were recorded and compared to the ones obtained for NaBH_4_ AgNPs and active carbon (Figure 4).

The intensity of active carbon peaks was weak so its contribution on the spectra of extracts absorbed on it was considered minimal (or non-interfering).

The spectra were recorded in the 3600–400 cm^−1^ range. They all exhibited a strong to medium intensity band at ca. 3400 cm^−1^ which is attributed to the O-H stretching vibrations, v(OH), of alcohols, phenols, and carbohydrates, or even water in the case of extracts [7,28,30]. However, a shift in the peak maximum to lower wavenumbers was recorded in the spectra of the extracts and extract AgNPs (Supplementary Materials Figure S10), possibly due to the appearance of an amine (N-H) bond [31].

The weak bands at ca. 2900 cm^−1^ were due to the C-H stretching vibrations, v(C-H) [32]. The wavenumbers indicate the existence of predominantly aliphatic C-H bonds. The band detected at 2400 cm^−1^ can only be assigned to one of the antisymmetric stretching vibrations of CO_2_ which was present in the instrument’s room and it is adsorbed by the samples [33]. 

The band at 1650 cm^−1^ can be attributed to C=O of carboxylic acids (COOH) as well as to N-H bond of primary amines [31]. 

The aromatic content of the samples is indicated from the appearance of weak bands in the 1600–1450 cm^−1^ region attributed to the carbon-carbon stretching vibrations [30,32]. 

The band at 1430 cm^−1^ evident in all extracts’ spectra can be assigned to the CH_2_-scissoring stretching vibration at the planar region [31]. A weak band at 1385 cm^−1^ in the extracts’ spectra is assigned to the O-H in-plane bending vibration of phenols [34]. However, the specific band was amplified in all extract reduced AgNPs spectra, suggesting that the capping agent of the nanoparticles possesses aromatic amine group with linkages between amino acid residues [27,28,31].

Several C-N vibration peaks were present in the range 1200–900 cm^−1^ of the spectra of extracts and extract reduced AgNPs [31].

The bands at ca. 1100 cm^−1^ (assigned to a C-H in-plane deformation mode) and 1046 cm^−1^ (assigned to C-O stretching vibrations from reducing sugars like glucose, fructose and sucrose) was more prominent for the extract AgNPs implying that those molecules participate in the reduction of silver ions [34].

Finally, the weak bands below 1000 cm^−1^ are assigned to various C-H out-of-plane deformations [27,28,32,35].

### 3.4. Total Phenolic (TPC) and Total Flavonoid (TFP) Content

The TPC and TFP of the extracts and the supernatants after AgNPs synthesis were analyzed and the results are summarized in Table 3 as gallic acid and quercetin equivalents, respectively. The reduction of total phenols and total flavonoids in the extracts was calculated in order to determine the amount of phenols and flavonoids that were used for the synthesis of AgNPs or were linked on their surfaces.

The reduction of total phenols for the sea buckthorn and sage extracts was higher reaching up to 66.02% ± 3.50% and 37.00% ± 4.42% respectively. The smallest reduction was noted for dittany extract, 12.36% ± 4.28%. 

The reduction of TFC was lower compared to the reduction of TPC. The highest reduction was calculated for the sage and calendula extracts (26.23% ± 3.28% and 24.16% ± 6.83% respectively) while for sea buckthorn and dittany extracts the reduction was slightly lower (18.56% ± 3.61 and 15.32% ± 7.25, respectively).

The reduction of total phenols for the sea buckthorn and sage extracts was higher, reaching up to 66.02% ± 3.50% and 37.00% ± 4.42% respectively. The smallest reduction was noted for dittany extract, 12.36% ± 4.28%. 

The reduction of TFC was lower compared to the reduction of TPC. The highest reduction was calculated for the sage and calendula extracts (26.23% ± 3.28% and 24.16% ± 6.83% respectively) while for sea buckthorn and dittany extracts, the reduction was slightly lower (18.56% ± 3.61% and 15.32% ± 7.25%, respectively).

### 3.5. Antioxidant Activity

The IC50s of the AgNPs and the respective extracts are summarized in Table 3. It appears that the sea buckthorn, sage, and NaBH_4_ AgNPs had the strongest antioxidant activity. On the contrary, dittany and calendula AgNPs had very low antioxidant activity compared to the rest. While dittany extract was found to be the most antioxidant, it did not generate very potent antioxidant AgNPs. Based on these results, it can be assumed that the antioxidant activity of the AgNPs is connected to the reduction of TPC and TFC during the reaction of AgNPs, i.e., to the amount of phenolic or flavonoid constituents of the extracts that were adsorbed on nanoparticles surface, and not to the antioxidant activity of the original extracts. Finally, the high antioxidant activity of sea buckthorn and sage AgNPs may be due to the phenols and flavonoids that may be present on the capping layer surrounding AgNPs surfaces. 

### 3.6. In Vitro Permeability Studies

#### 3.6.1. Transdermal Penetration of AgNPs

TXRF analysis of the samples of PBS that were collected from the donor compartment of the Franz cells detected inorganic elements like the ingredients of PBS, while Ag was not found in any sample (Supplementary Materials Table S4). This result indicates that the AgNPs did not permeate through the intact skin, rendering them safe for topical application on intact skin barrier.

#### 3.6.2. Dermal Penetration of AgNPs

The results of the TXRF analysis of the Ag content in the SC that was removed by tape stripping and the calculation of the percentage of the initial amount that was applied are summarized in Table 4. The Ag content in SC did not differ significantly between the samples ranging from 1.76 ± 0.52 μg/cm^2^ for NaBH_4_ AgNPs to 0.71 ± 0.04 μg/cm^2^ for sea buckthorn AgNPs. The percentage of the initially applied Ag that was found in the SC ranged between 11.26% ± 3.36% to 4.55% ± 0.27% for NaBH_4_ and sea buckthorn AgNPs, respectively. These results indicate that the cutaneous application of the prepared AgNPs for 4 h led to incorporation in the SC of about 10% of the applied amount, while the AgNPs did not penetrate the intact skin.

## 4. Discussion

AgNPs were synthesized using green chemistry employing four commercially available hydroglycolic extracts of dittany, sage, sea buckthorn, and calendula. The impact of light, heating, and reaction time on their preparation was studied and optimal conditions were decided for every case. 

Rahman et al. (2019) described AgNPs synthesis as a three-step procedure. In the first step that it takes place in the dark without the need of photon energy, Ag ions are adsorbed on extracellular polymeric substances (EPS) [36]. The second step takes place only when assisted by a source of energy (i.e., photons) that enables the conversion of Ag ions to AgNPs, while in the third step, the AgNPs are stabilized by EPS by forming the capping halo around their surfaces.

Although it is reported that the temperature is a factor that determines the formation and growth, shape, size, and size distribution of AgNPs [37], our results suggest that this is not the only important factor. Other parameters like the presence of light, reaction time and rate, and the extract used should be taken into consideration when designing AgNPs production using plant extracts. 

In particular, the reaction of AgNPs synthesis was achieved under light at room temperature for all the extracts, and heating was needed only when sea buckthorn extract was used. In the case of calendula AgNPs, in which the high content of Cl ions was hindering the reaction of Ag ion reduction, the determining factor was time. This conclusion is based on the fact that when the reaction was allowed to take place for 24 h under light, the result was the same either at RT or with heating at 50–60 °C, as was demonstrated by the absorbance intensity of UV-Vis spectra of nanoparticle synthesized with calendula. Furthermore, the presence of Cl ions in the calendula extract led to the formation of Ag-halides hindering the synthesis of AgNPs and affecting their physicochemical characteristics and long-term stability. According to these results, the composition of the extract and the presence of ions like Cl- that may hinder the synthesis of AgNPs should be taken into consideration when designing their synthesis and selecting the reducing plant extract. Additionally, the chosen parameters may affect the morphology, the physicochemical characteristics, and long-term stability of the synthesized AgNPs. The round-shaped calendula AgNPs were larger in comparison to the others, as was revealed by TEM, and lacked rod-shaped or triangular AgNPs. This fact may be attributed to the slow rate of calendula AgNPs synthesis. It has been reported that the time needed for the Ag+ reduction affects the packaging of unit lattices during Ag crystal formation as slow reduction rate of Ag+ may lead to loose packing of unit lattices affecting thus the type of Ag crystal and consecutively the shape of AgNPs [22,27]. Additionally, calendula AgNPs were highly aggregated, a fact that may indicate destabilization with storage time.

The contribution of phenolic compounds and carbohydrates such as reducing sugars in the reduction of Ag ion is demonstrated by the shift of their FTΙR peaks.

Bioorganic material participating in a capping halo that stabilizes the AgNPs affects their long-term stability. According to FTΙR results, it seems that bioorganic substances such as carbohydrates, but also phenolic and flavonoid compounds, have participated in the reduction of Ag ion and AgNPs formation. Although the TPC and TFC in the capping substance could not be directly measured it was estimated indirectly by monitoring their reduction during AgNPs synthesis and the antioxidant capacity of the prepared AgNPs. According to our results, we may conclude that phenolic and flavonoid compounds participated in Ag ions reduction and in the capping substance that stabilized AgNPs, and are also accountable for their antioxidant capacity. The importance of TPC and TFC of the extracts in the AgNPs formation is described in detail in the literature [38]. 

The results on dermal and transdermal penetration of AgNPs are in accordance with Tak et al. (2015) [22]. In that work, the authors reported that in vitro transdermal NPs penetration was obvious after 8 h and peaked at 12 h after cutaneous application of the AgNPs. Additionally, they related the shape of nanoparticles to the rate of penetration pointing out that the triangular and spherical AgNPs demonstrate the slowest penetration rate compared to the rod-shaped ones.

Rod-shaped NPs were able to reach the dermis where they could accumulate, rapidly increasing the possibility of systemic toxicity. Spherical NPs could reach up to the epidermis while triangular NPs on the other hand could not reach beyond stratum corneum demonstrating low penetration capabilities that could be attributed to the predominant (111) facets. This fact, in combination with their highest degree of bactericidal activity, makes them ideal candidates for topical applications in comparison to the spherical or rod-shaped NPs.

Although the shape of the prepared AgNPs as revealed by TEM was mainly spherical in some cases (like sage and dittany NPs), triangular NPs were present while rod-shaped AgNPs were rare (Figure 1). This fact may explain the relatively low transdermal absorption of the nanoparticles after 4 h of application (Table IV).

Although in previous works permeation through intact skin was detected as soon as 1 h after application [39], recently it was demonstrated that the degree and penetration depth of AgNPs is controlled by factors such as size, shape, and type of capping material of AgNPs [22,40,41].

Kraeling et al., 2018 [41] have reported that most of the AgNPs (about 80% of the applied dose) remained on the skin surface while at the receptor fluid, AgNPs concentration was below Limit of detection (LOD), 24 h after application of their aqueous dispersion on the donor chamber of Franz cells.

## 5. Conclusions

In order to investigate the impact of the plant extracts used as reducing agents to the physicochemical characteristics and properties of the synthesized AgNPs, we compared four plant extracts regarding their influence on the reaction conditions, characteristics, and colloidal stability of AgNPs. Our results demonstrated the importance of plant extract selection to the reaction conditions that should be applied for green synthesis of AgNPs. Moreover, because the AgNPs of this study did not exhibit skin permeation 4 h after application, we can conclude that when applied to intact skin for a short time, they can act topically without the risk of systemic absorption.

Previous reports show that triangular or spherical AgNPs have slow penetration capabilities in stratum corneum and low toxicity compared to rod-shaped nanoparticles [22]. The AgNPs synthesized using dittany or sage extracts produced mainly spherical AgNPs with an appreciable number of triangular ones. 

These results may guide further investigations for optimizing conditions of formation of desired shaped AgNPs using selected extracts and are expected to lead to novel materials with enhanced properties and low potential of systemic toxicity.

## Figures and Tables

**Table 1:** Physicochemical characterization of AgNPs (mean values ± SD).

Type of AgNPs	Size (nm)	PDI	ζ-Potential (mV)	Zdev
Dittany	79.00 ± 0.50	0.382 ± 0.022	−30.90 ± 0.36	8.48 ± 0.66
Sage	93.06 ± 1.58	0.366 ± 0.003	−28.77 ± 0.38	7.12 ± 0.88
Sea buckthorn	137.5 ± 0.76	0.269 ± 0.006	−29.77 ± 0.40	5.43 ± 1.28
Calendula	364.6 ± 13.09	0.408 ± 0.018	−31.50 ± 0.72	4.18 ± 0.22
NaBH_4_	76.90 ± 0.14	0.441 ± 0.008	−26.87 ± 0.29	7.56 ± 0.36

**Table 2:** Concentration (ppm) of Ag and Cl in AgNPs and extracts (mean values ± SD).

Sample		AgNPs		Extracts
	Element	Ag	Cl	Cl
Extract				
Dittany		821.7 ± 171.6	29.5 ± 5.3	19.0 ± 10.3
Sage		825.5 ± 84.6	25.4 ± 2.0	3.8 ± 2.9
Sea buckthorn		670.7 ± 30.8	22.6 ± 0.5	0.2 ± 0.0
Calendula		863.1 ± 33.1	166.6 ± 2.7	127.4 ± 16.6
NaBH_4_		315.66 ± 37.3	6.14 ± 0.6	-

**Table 3:** Total phenolic and flavonoid content and antioxidant activity of AgNPs and the extracts before and after the AgNPs synthesis (mean values ± SD).

Sample	Extracts (before Reaction)	Supernatants (after Reaction)	% Reduction
Total phenolic content (gallic acid equivalents mg/100 g of dry plant)			
Dittany	1.57 ± 0.07	1.47 ± 0.06	12.36 ± 4.28
Sage	1.22 ± 0.07	0.80 ± 0.02	37.00 ± 4.42
Sea buckthorn	1.24 ± 0.07	0.43 ± 0.01	66.02 ± 3.50
Calendula	0.59 ± 0.04	0.45 ± 0.01	24.15 ± 6.64
Dittany	1.57 ± 0.07	1.47 ± 0.06	12.36 ± 4.28
Total flavonoid content (quercetin equivalents mg/100 g of dry plant)			
Dittany	1.47 ± 0.09	1.39 ± 0.16	15.32 ± 7.25
Sage	1.45 ± 0.12	1.16 ± 0.10	26.23 ± 3.28
Sea buckthorn	0.75 ± 0.05	0.62 ± 0.04	18.56 ± 3.61
Calendula	0.66 ± 0.06	0. 58 ± 0.10	24.16 ± 6.83
Dittany	1.47 ± 0.09	1.39 ± 0.16	15.32 ± 7.25
Antioxidant activity of AgNPs, of the extracts, and the ascorbic acid expressed as IC50 (mg/mL) (mean values ± SD)			
Sample	**Extracts**	**AgNPs**	
Dittany	11.11 ± 0.30	1.92 ± 0.13	
Sage	15.05 ± 0.49	0.77 ± 0.04	
Sea buckthorn	18.43 ± 0.94	0.61 ± 0.07	
Calendula	69.29 ± 1.83	2.08 ± 0.29	
NaBH_4_	-	0.69 ± 0.08	
Ascorbic acid	0.24 ± 0.00		

**Table 4:** Ag detected in the stratum corneum.

AgNPs	Ag Concentration (μg/cm^2^)	% of Initially Applied Ag
Dittany	1.58 ± 0.59	10.14 ± 3.80
Sage	1.42 ± 0.29	9.09 ± 1.83
Sea buckthorn	0.71 ± 0.06	4.55 ± 0.38
Calendula	1.54 ± 0.25	9.83 ± 1.61
NaBH_4_	1.76 ± 0.52	11.26 ± 3.35

## Supplementary Materials

The following are available online at https://www.mdpi.com/1999-4923/12/12/1244/s1, Table S1. Physicochemical characterization of AgNPs prepared under different conditions Table S2. Stability study of AgNPs Table S3. SAED patterns of AgNPs Table S4. Concentration (μg/mL) of the elements in the receptor compartment of Franz cells (mean ± SD) Figure S1. UV/Vis spectrum and absorbance peak of (a) dittany, (b) sage, (c) sea buckthorn, (d) calendula, and (e) NaBH_4_ AgNPs. Figure S2. UV/Vis spectrum of (a) dittany, (b) sage, (c) sea buckthorn, and (d) calendula AgNPs synthesized at RT in the dark (I) or under light (II) Figure S3. Color change of the dispersions during the reaction under light for the synthesis of AgNPs derived from the extracts (a) dittany, (b) sea buckthorn, (c) sage, and (d) calendula at RT (I) or with heating at 50 °C (II) after 1, 3, 5, and 24 h. Figure S4. UV/Vis of the calendula AgNPs synthesized under light and heating at 50–60 °C for 2 h. Figure S5. Study of the synthesis of dittany AgNPs monitoring the intensity of the absorbance of the reaction mixture (a) without or (b) with heating, (c) the alteration of absorbance over time, (d) the mean size (column) and PDI (line), and (e) the ζ-potential of the nanoparticles prepared without (■−) or with (■−) heating at predetermined time points. Figure S6. Study of the synthesis of Sage AgNPs monitoring the intensity of the absorbance of the reaction mixture (a) without or (b) with heating, (c) the alteration of absorbance over time, (d) the mean size (column) and PDI (line), and (e) the ζ-potential of the nanoparticles prepared without (■−) or with (■−) heating at predetermined time points. Figure S7. Study of the synthesis of sea buckthorn AgNPs monitoring the intensity of the absorbance of the reaction mixture (a) without or (b) with heating, (c) the alteration of absorbance over time, (d) the mean size (column) and PDI (line), and (e) the ζ-potential of the nanoparticles prepared without (■−) or with (■−) heating at predetermined time points. Figure S8. Study of the synthesis of calendula AgNPs monitoring the intensity of the absorbance of the reaction mixture (a) without or (b) with heating, (c) the alteration of absorbance over time, (d) the mean size (column) and PDI (line), and (e) the ζ-potential of the nanoparticles prepared without (■−) or with (■−) heating at predetermined time points. Figure S9. Comparison of the alteration of the UV/Vis absorbance of (a) dittany, (b) sage, (c) sea buckthorn and (d) calendula AgNPs at every time point with (■) or without (■) heating (*: *p* < 0.05 **: *p* < 0.005). Figure S10. FTIR spectra of calendula AgNPs (--), calendula extract (--), and NaBH_4_ AgNPS (--), in the spectral area (a) 2500–4000 cm^−1^ and (b) 500–2000 cm^−1^. The shift of the main peak maximum is shown.

## References

[1] Sharma V.K., Yngard R.A., Lin Y. (2009). Silver nanoparticles: Green synthesis and their antimicrobial activities. Adv. Colloid Interface Sci..

[2] Abou EL-Nour K.M.M., Eftaiha A., Al-Warthan A., Ammar R.A.A. (2010). Synthesis and applications of silver nanoparticles. Arab. J. Chem..

[3] Balzarro M., Rubilotta E., Trabacchin N., Soldano A., Cerrato C., Migliorini F., Mancini V., Pastore A.L., Carbone A., Cormio L. (2020). Early and Late Efficacy on Wound Healing of Silver Nanoparticle Gel in Males after Circumcision. J. Clin. Med..

[4] Kokura S., Handa O., Takagi T., Ishikawa T., Naito Y., Yoshikawa T. (2010). Silver nanoparticles as a safe preservative for use in cosmetics. Nanomedicine.

[5] Kuppusamy P., Yusoff M.M., Maniam G.P., Govindan N. (2016). Biosynthesis of metallic nanoparticles using plant derivatives and their new avenues in pharmacological applications—An updated report. Saudi Pharm. J..

[6] Durán N., Marcato P.D., De Souza G.I.H., Alves O.L., Esposito E. (2007). Antibacterial effect of silver nanoparticles produced by fungal process on textile fabrics and their effluent treatment. J. Biomed. Nanotechnol..

[7] Yu D.G. (2007). Formation of colloidal silver nanoparticles stabilized by Na -poly(γ-glutamic acid)-silver nitrate complex via chemical reduction process. Colloids Surf. B Biointerfaces.

[8] Navaladian S., Viswanathan B., Viswanath R.P., Varadarajan T.K. (2006). Thermal decomposition as route for silver nanoparticles. Nanoscale Res. Lett..

[9] Sreeram K.J., Nidhin M., Nair B.U. (2008). Microwave assisted template synthesis of silver nanoparticles. Bull. Mater. Sci..

[10] Starowicz M., Stypuła B., Banaś J. (2006). Electrochemical synthesis of silver nanoparticles. Electrochem. Commun..

[11] Fahimirad S., Ajalloueian F., Ghorbanpour M. (2019). Synthesis and therapeutic potential of silver nanomaterials derived from plant extracts. Ecotoxicol. Environ. Saf..

[12] Otari S.V., Patil R.M., Nadaf N.H., Ghosh S.J., Pawar S.H. (2012). Green biosynthesis of silver nanoparticles from an actinobacteria *Rhodococcus* sp.. Mater. Lett..

[13] Mittal A.K., Chisti Y., Banerjee U.C. (2013). Synthesis of metallic nanoparticles using plant extracts. Biotechnol. Adv..

[14] Banne S.V., Patil M.S., Kulkarnic R.M., Patil S.J. (2017). Synthesis and Characterization of Silver Nano Particles for EDM Applications. Mater. Today Proc..

[15] Pinto V.V., Ferreira M.J., Silva R., Santo H.A., Silva F., Pereira C.M. (2010). Long time effect on the stability of silver nanoparticles in aqueous medium: Effect of the synthesis and storage conditions. Colloids Surf. A Physicochem. Eng. Asp..

[16] Schneider C.A., Rasband W.S., Eliceiri K.W. (2012). NIH Image to ImageJ: 25 years of image analysis. Nat. Methods.

[17] Folin O., Ciocalteu V. (1927). On tyrosinase and trytophane determinations in proteins. J. Biol. Chem..

[18] Ainsworth E., Gillepsie K. (2007). Estimation of total phenolic content and other oxidation substrates in plant tissues using Folin- Ciocalteu reagent. Nat. Protoc..

[19] Woisky R.G., Salatino A. (1998). Analysis of Propolis: Some Parameters and Procedures for Chemical Quality Control. J. Apicult. Res..

[20] Brand-Williams W., Cuvelier M.E., Berset C. (1995). Use of a free radical method to evaluate anioxidant activity. LWT Food Sci. Technol..

[21] Kim D.O., Lee K.W., Lee H.J., Lee C.Y. (2002). Vitamin C equivalent antioxidant capacity (VCEAC) of phenolic phytochemicals. J. Agric. Food Chem..

[22] Tak Y.K., Pal S., Naoghare P.K., Rangasamy S., Song J.M. (2015). Shape-Dependent Skin Penetration of Silver Nanoparticles: Does It Really Matter?. Sci. Rep..

[23] Ramesh A.V., Devi D.R., Battu G., Basavaiah K. (2018). A facile plant mediated synthesis of silver nanoparticles using an aqueous leaf extract of *Ficus hispida* Linn. F. for catalytic. antioxidant and antibacterial applications. S. Afr. J. Chem. Eng..

[24] Manikandan R., Manikandan B., Raman T., Arunagirinathan K., Prabhu N.M., Jothi Basu M., Perumal M., Palanisamy S., Munusamy A. (2015). Biosynthesis of silver nanoparticles using ethanolic petals extract of *Rosa indica* and characterization of its antibacterial. Anticancer and anti-inflammatory activities. Spectrochim. Acta A Mol. Biomol. Spectrosc..

[25] Firoozi S., Jamzad M., Yari M. (2016). Biologically synthesized silver nanoparticles by aqueous extract of *Satureja intermedia* C.A. Mey and the evaluation of total phenolic and flavonoid contents and antioxidant activity. J. Nanostruct. Chem..

[26] Bhaduri G.A., Little R., Khomane R.B., Lokhande S.U., Kulkarni B.D., Mendis B.G., Siller L. (2013). Green synthesis of silver nanoparticles using sunlight. J. Photochem. Photobiol. A.

[27] Mishra P.M., Sahoo S.K., Naik G.K., Parida K. (2015). Biomimetic synthesis, characterization and mechanism of formation of stable silver nanoparticles using *Averrhoa carambola* L. leaf extract. Mater. Lett..

[28] Zhan G., Huang J., Lin L., Lin W., Emmanuel K., Li Q. (2011). Synthesis of gold nanoparticles by *Cacumen Platycladi* leaf extract and its simulated solution: Toward the plant-mediated biosynthetic mechanism. J. Nanopart. Res..

[29] Baghizadeh A., Ranjbar S., Gupta V.K., Asif M., Pourseyedi S., Javad Karimi M., Mohammadinejad R. (2015). Green synthesis of silver nanoparticles using seed extract of Calendula officinalis in liquid phase. J. Mol. Liq..

[30] Keling C., Kenan X., Qin L., Lijun D., Zhiqiang F., Huanhuan X., Lu X. (2017). Fabrication of core–shell Ag@pDA@Hap nanoparticles with the ability for controlled release of Ag+ and superior hemocompatibility. RSC Adv..

[31] Gudikandula K., Metuku R.P., Burra S., Nidadavolu S.V., Pabba S.K., Alha S.C. (2015). Fungus-Mediated Synthesis of Silver Nanoparticles and Their Activity against Gram Positive and Gram Negative Bacteria in Combination with Antibiotics. Walailak J. Sci. Technol..

[32] Bellamy L.J. (1966). The Infra-red Spectra of Complex Molecules.

[33] Nakamoto K. (1986). Infrared and Raman Spectra of Inorganic and Coordination Compounds.

[34] Silverstein R.M., Bassler G.C., Morrill T.C. (1981). Spectrometric Identification of Organic Compounds.

[35] Prakash P., Gnanaprakasam P., Emmanuel R., Arokiyaraj S., Saravanan M. (2013). Green synthesis of silver nanoparticles from leaf extract of *Mimusops elengi*, Linn. for enhanced antibacterial activity against multi drug resistant clinical isolates. Colloids Surf. B Biointerfaces.

[36] Rahman A., Kumar S., Bafana A., Lin J., Dahoumane S.A., Jeffryes C. (2019). A Mechanistic View of the Light-Induced Synthesis of Silver Nanoparticles Using Extracellular Polymeric Substances of *Chlamydomonas reinhardtii*. Molecules.

[37] Jiang X.C., Chen W.M., Chen C.Y., Xiong S.X., Yu A.B. (2011). Role of Temperature in the Growth of Silver Nanoparticles Through a Synergetic Reduction Approach. Nanoscale Res. Lett..

[38] Anjum S., Abbasi B.H. (2016). Thidiazuron-enhanced biosynthesis and antimicrobial efficacy of silver nanoparticles via improving phytochemical reducing potential in callus culture of *Linum usitatissimum* L.. Int. J. Nanomed..

[39] Larese F.F., D’Agostin F., Crosera M., Adami G., Renzi N., Bovenzi M., Maina G. (2009). Human skin penetration of silver nanoparticles through intact and damaged skin. Toxicology.

[40] Ezealisiji K.M., Okorie H.N. (2018). Size-dependent skin penetration of silver nanoparticles: Effect of penetration enhancers. Appl. Nanosci..

[41] Kraeling M.E.K., Topping V.D., Keltner Z.K., Belgrave K.R., Bailey K.D., Gao X., Yourick J.J. (2018). In vitro percutaneous penetration of silver nanoparticles in pig and human skin. Regul. Toxicol. Pharmacol..

