# Supplementary material for: Effect of Plant Extracts on the Characteristics of Silver Nanoparticles for Topical Application
Source: Pharmaceutics. 2020 Dec 21;12(12):1244. doi: 10.3390/pharmaceutics12121244 (PMC7767050; doi:10.3390/pharmaceutics12121244)
Supplement: Supplementary file 1 [file pharmaceutics-12-01244-s001.pdf]

pharmaceutics

Supplementary Materials

Article

Effect of Plant Extracts on the Characteristics of Silver Nanoparticles for Topical Application

Ioanna K. Siakavella [1], Fotini Lamari [2], Dimitrios Papoulis [3], Malvina Orkoula [4], Patroula Gkolfi [5], Michail Lykouras [4], Konstantinos Avgoustakis [1] and Sophia Hatziantoniou [1,*]

[1] Laboratory of Pharmaceutical Technology, Department of Pharmacy, School of Health Sciences, University of Patras, 26504 Patras, Greece; ioanna.siakav@gmail.com (I.K.S.); avgoust@upatras.gr (K.A.); sohatzi@upatras.gr (S.H.)
[2] Laboratory of Pharmacognosy & Chemistry of Natural Products Department of Pharmacy, School of Health Sciences, University of Patras, 26504 Patras, Greece; flam@upatras.gr
[3] Department of Geology, University of Patras, 26504 Patras, Greece; papoulis@upatras.gr
[4] Laboratory of Instrumental Pharmaceutical Analysis, Department of Pharmacy, School of Health Sciences, University of Patras, 26504 Patras, Greece; malbie@upatras.gr (M.O.); michalislyk@gmail.com (M.L.)
[5] Department of Chemistry, University of Patras, 26504 Patras, Greece; patroula.gkolfi@gmail.com
* Correspondence: sohatzi@upatras.gr

Table S1. Physicochemical characterization of AgNPs prepared under different conditions

Conditions	light	No				No				Yes			
	Heat	No				Yes				Yes			
**Ag NP**	**Property**	**size (nm)**	**PDI**	**ζ−potential (mV)**	**Zdev**	**size (nm)**	**PDI**	**ζ−potential (mV)**	**Zdev**	**size (nm)**	**PDI**	**ζ−potential (mV)**	**Zdev**
**Dittany**		189.60 ± 0.93	0.25 ± 0.01	−25.40 ± 0.82	4.47 ± 0.04	112.45 ± 15.49	0.25 ± 0.00	−24.83 ± 0.19	6.27 ± 0.23	117.40 ± 3.54	0.39 ± 0.02	−18.47 ± 0.38	5.16 ± 0.87
**Sage**		-	-	-	-	161.75 ± 0.32	0.32 ± 0.09	−27.53 ± 9.33	6.48 ± 1.5	150.50 ± 4.81	0.39 ± 0.03	−21.85 ± 1.15	5.19 ± 0.11
**Sea buckthorn**		288.70 ± 3.83	0.26 ± 0.01	−20.80 ± 0.44	5.19 ± 1.24	209.45 ± 4.64	0.24 ± 0.05	−22.30 ± 3.21	5.77 ± 0.39	199.30 ± 0.00	0.23 ± 0.01	−19.22 ± 0.82	6.09 ± 0.46
**Calendula**		-	-	-	-	292.75 ± 36.6	0.28 ± 0.07	−28.67 ± 1.18	4.35 ± 0.39	-	-	-	-

Table S2. Stability study of AgNPs

Time (d)	Size (nm)	PDI	ζ–potential (mV)	Zdev
	**Dittany**			
**1**	81.58 ± 9.54	0.302 ± 0.026	−29.26 ± 0.76	8.75 ± 1.32
**8**	81.31 ± 8.33	0.303 ± 0.026	−21.78 ± 1.03	7.48 ± 1.71
**15**	78.72 ± 7.81	0.300 ± 0.020	−23.79 ± 1.54	8.06 ± 1.39
**30**	79.27 ± 7.84	0.327 ± 0.059	−26.76 ± 2.57	7.99 ± 1.84
**60**	80.35 ± 7.34	0.294 ± 0.006	−23.19 ± 4.08	7.29 ± 1.41
**90**	77.10 ± 7.79	0.310 ± 0.04	−22.99 ± 0.50	8.01 ± 1.37
**120**	79.32 ± 8.09	0.320 ± 0.04	−27.28 ± 1.69	8.36 ± 1.13
	**Sage**			
**1**	110.79 ± 14.41	0.347 ± 0.071	−28.10 ± 1.74	6.97 ± 1.57
**8**	109.33 ± 10.34	0.318 ± 0.013	−24.27 ± 1.90	5.56 ± 0.75
**15**	112.84 ± 11.83	0.363 ± 0.058	−23.18 ± 1.27	5.53 ± 1.00
**30**	112.87 ± 5.22	0.322 ± 0.022	−25.17 ± 2.39	5.94 ± 1.21
**60**	115.30 ± 5.51	0.354 ± 0.045	−24.22 ± 3.56	5.07 ± 0.30
**90**	114.33 ± 6.21	0.360 ± 0.039	−23.20 ± 1.48	5.60 ± 0.94
**120**	110.77 ± 6.20	0.348 ± 0.029	−22.90 ± 3.19	5.61 ± 0.40
	**Sea Buckthorn**			
**1**	134.13 ± 6.10	0.281 ± 0.006	−25.63 ± 0.87	4.99 ± 0.18
**8**	130.67 ± 4.74	0.291 ± 0.017	−23.20 ± 1.10	5.47 ± 0.23
**15**	130.57 ± 4.29	0.301 ± 0.038	−23.87 ± 1.17	5.02 ± 0.38
**30**	133.03 ± 2.60	0.300 ± 0.033	−26.19 ± 4.13	5.25 ± 0.78
**60**	137.23 ± 6.14	0.270 ± 0.016	−27.60 ± 3.11	4.81 ± 0.22
**90**	130.00 ± 2.54	0.282 ± 0.010	−23.17 ± 2.31	4.73 ± 0.34
**120**	133.30 ± 12.66	0.285 ± 0.020	−26.16 ± 5.44	4.98 ± 0.31
	**Calendula**			
**1**	285.63 ± 16.38	0.252 ± 0.031	−28.96 ± 1.12	5.47 ± 1.45
**8**	309.23 ± 40.57	0.289 ± 0.044	−31.23 ± 5.46	4.85 ± 0.54
**15**	349.47 ± 32.19	0.333 ± 0.082	−30.66 ± 2.18	4.35 ± 0.08
**30**	311.87 ± 52.29	0.303 ± 0.078	−30.62 ± 3.05	5.93 ± 1.99
**60**	272.93 ± 10.23	0.249 ± 0.016	−29.51 ± 2.86	4.62 ± 0.17
**90**	303.87 ± 18.35	0.373 ± 0.070	−29.54 ± 16.43	4.53 ± 2.84
**120**	336.77 ± 113.55	0.268 ± 0.056	−17.14 ± 22.25	4.22 ± 0.47
	**$NaBH_4$**			
**1**	142.85 ± 93.27	0.403 ± 0.054	−16.94 ± 14.04	5.88 ± 2.38
**8**	130.01 ± 45.10	0.342 ± 0.016	−22.90 ± 2.17	8.76 ± 4.06
**60**	138.30 ± 67.74	0.398 ± 0.035	−22.61 ± 19.83	7.68 ± 2.64
**90**	160.40 ± 22.91	0.551 ± 0.206	−17.05 ± 2.90	10.35 ± 0.24
**120**	148.90 ± 60.95	0.480 ± 0.169	−15.74 ± 7.82	6.11 ± 1.00

Table S3. SAED patterns of AgNPs.

d-spacing (nm)					Miller index
**Dittany**	**Sage**	**Sea buckthorn**	**Calendula**	**$NaBH_4$**	
0.2838	-		0.2804	-	Ag (101)
0.2400	0.2322	0.2398	0.2323	0.2359	Ag (111)
0.2088	0.2040	0.2043	-	0.204	Ag (200)
0.1453	0.1463	0.1467	0.1466	0.1446	Ag (220)
0.1234	0.1257	-	0.1244	0.1244	Ag (311)
	0.1915	0.1960	0.1975	-	AgCl (220)
	0.1603	-	0.1642	-	AgCl (222)

Table S4. Concentration (μg/ml) of the elements in the receptor compartment of Franz cells (mean ± SD)

Element	Dittany	Sage	Sea buckthorn	Calendula	$NaBH_4$
**P**	196.88 ± 16.94	189.77 ± 2.40	192.89 ± 20.69	195.19 ± 14.05	251.91 ± 24.81
**Cl**	3102.01 ± 50.38	3045.68 ± 141.84	3217.00 ± 122.83	3082.32 ± 179.31	3542.41 ± 314.80
**K**	104.61 ± 13.33	95.83 ± 1.40	91.41 ± 7.99	89.14 ± 6.59	116.53 ± 11.08
**Ca**	4.24 ± 0.62	2.54 ± 0.24	2.84 ± 0.57	2.24 ± 0.35	5.64 ± 1.64
**Mn**	0.02 ± 0.04	0.00 ± 0.00	0.00 ± 0.00	0.00 ± 0.01	0.01 ± 0.01
**Fe**	0.05 ± 0.03	0.16 ± 0.12	0.06 ± 0.03	0.03 ± 0.01	0.06 ± 0.06
**Cu**	0.01 ± 0.01	0.02 ± 0.01	0.01 ± 0.01	0.00 ± 0.00	0.00 ± 0.00
**Zn**	0.13 ± 0.05	0.23 ± 0.03	0.21 ± 0.09	0.12 ± 0.00	0.37 ± 0.05
**Br**	0.70 ± 0.09	0.67 ± 0.02	0.52 ± 0.05	0.40 ± 0.03	0.64 ± 0.03
**Ag**	0.00 ± 0.00	0.00 ± 0.00	0.00 ± 0.00	0.00 ± 0.00	0.00 ± 0.00

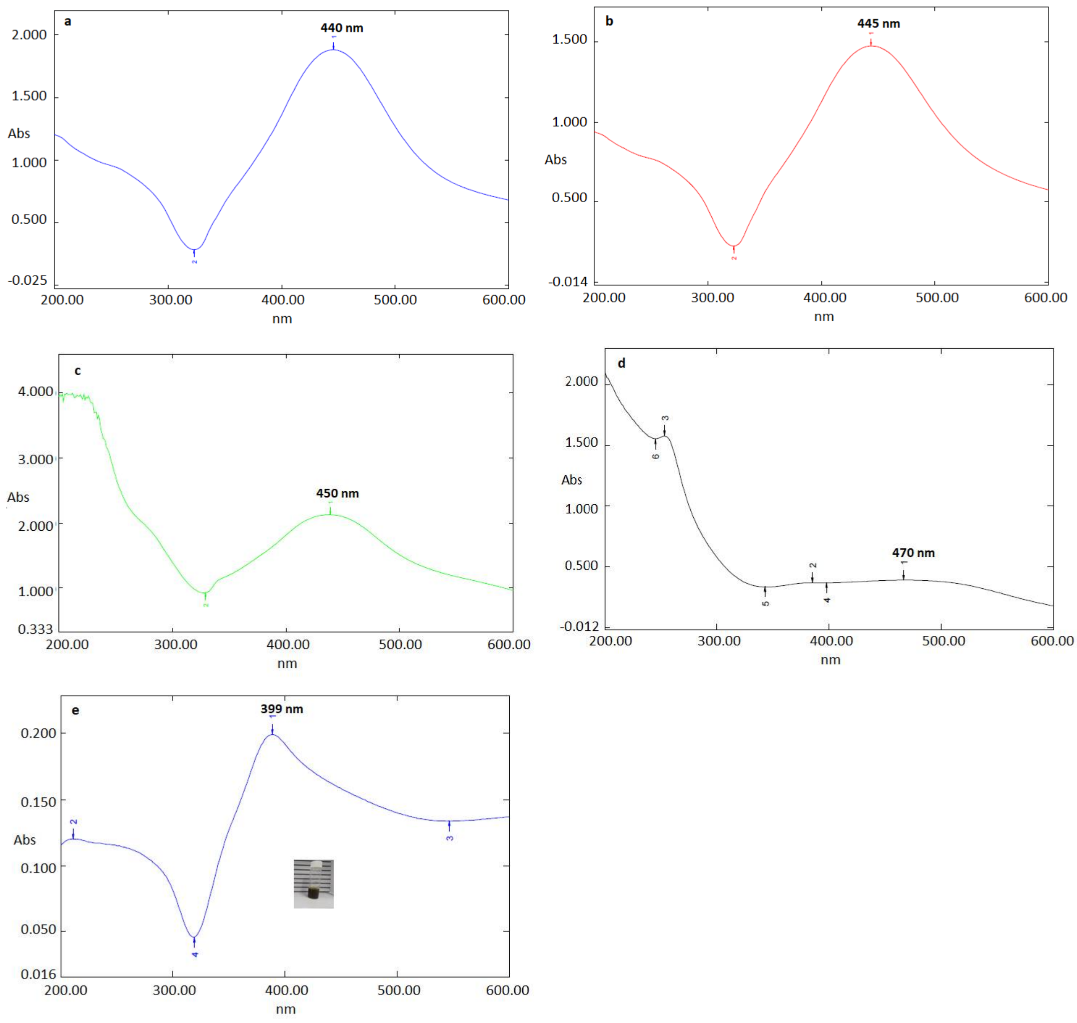

Figure S1. UV/Vis spectrum and absorbance peak of (a) Dittany, (b) Sage, (c) Sea buckthorn, (d) Calendula and (e) $NaBH_4$ AgNPs.

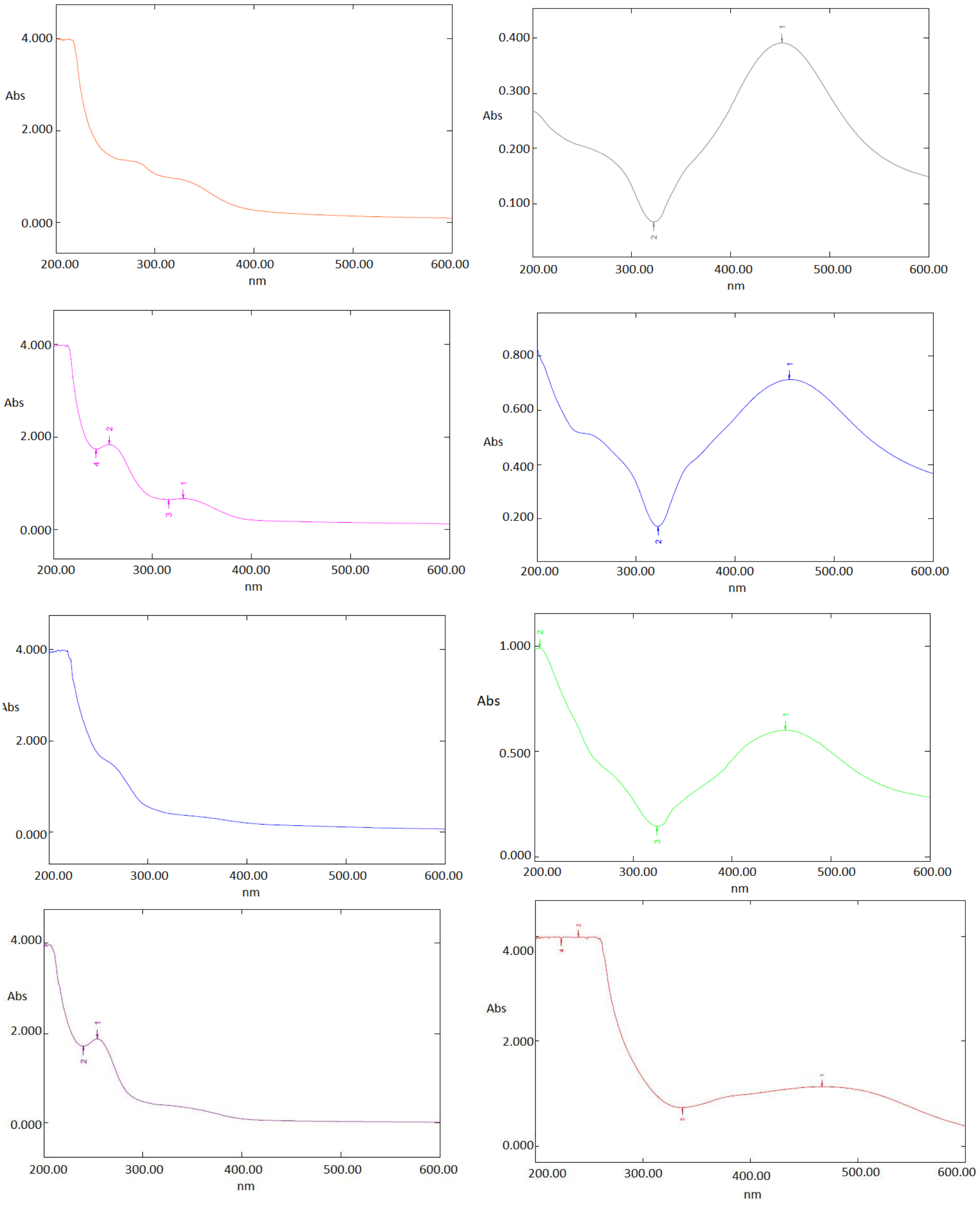

Figure S2. UV/Vis spectrum of (a) Dittany, (b) Sage, (c) Sea buckthorn and (d) Calendula AgNPs synthesized at RT in the dark (I) or under light (II)

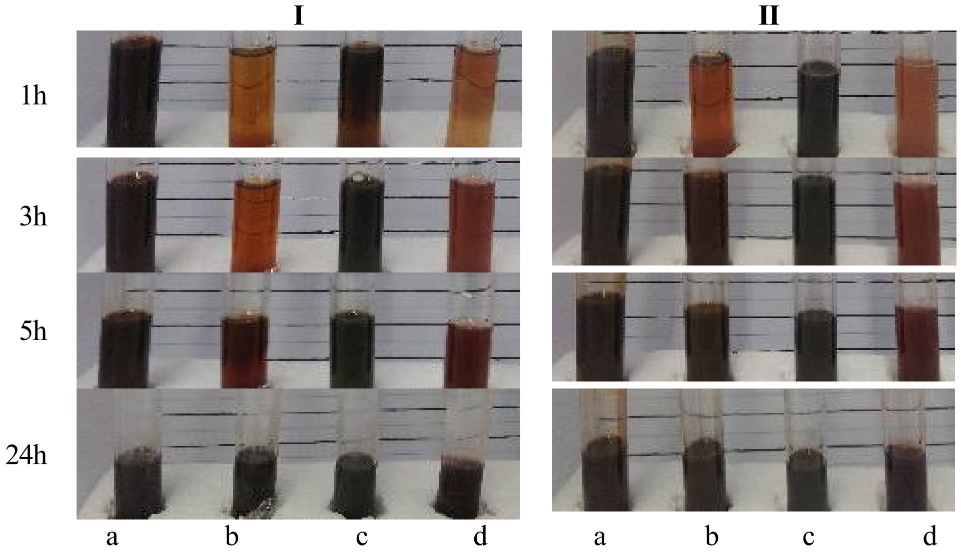

Figure S3. Color change of the dispersions during the reaction under light for the synthesis of AgNPs derived from the extracts (a) Dittany, (b) Sea buckthorn, (c) Sage, (d) Calendula at RT (I) or with heating at 50 °C (II) after 1, 3, 5 and 24h.

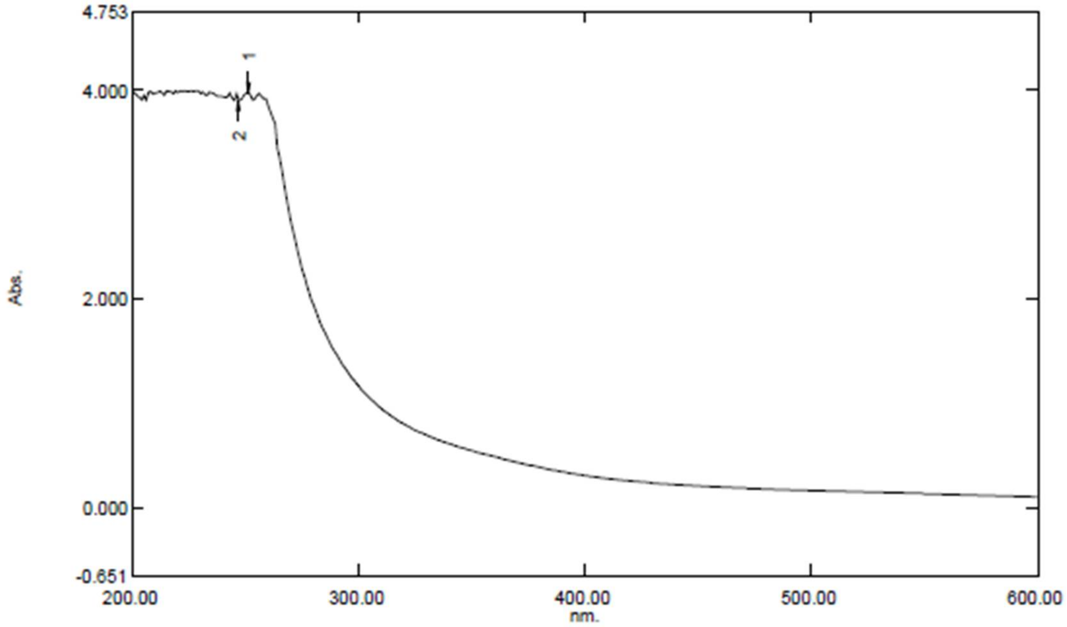

Figure S4. UV/Vis of the Calendula AgNPs synthesized under light and heating at 50–60°C for 2 h.

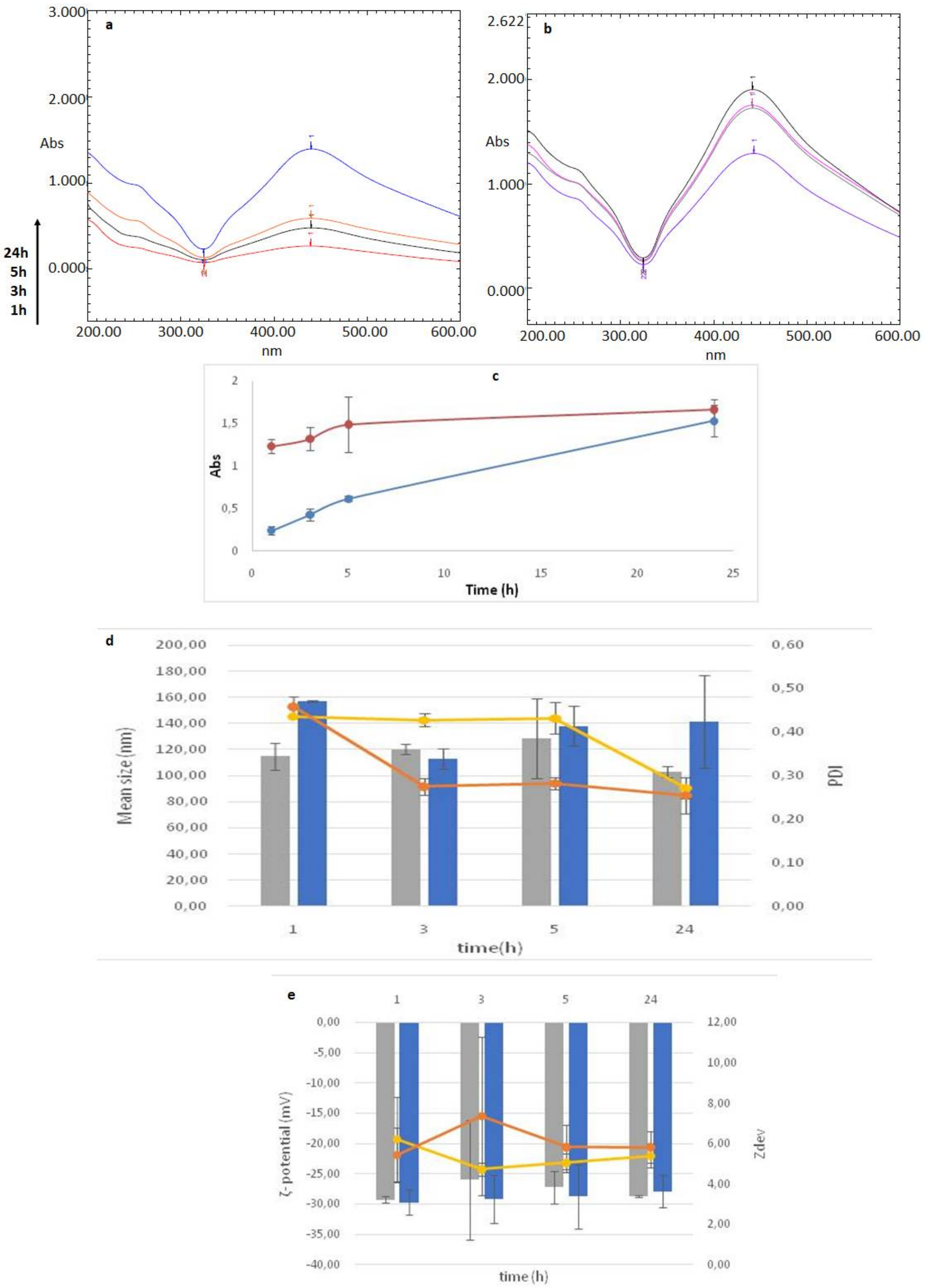

Figure S5. Study of the synthesis of Dittany AgNPs monitoring the intensity of the absorbance of the reaction mixture (a) without or (b) with heating, (c) the alteration of absorbance over time, (d) the mean size (column) and PDI (line) and (e) the ζ-potential of the nanoparticles prepared without (■—) or with (■—) heating at predetermined time points.

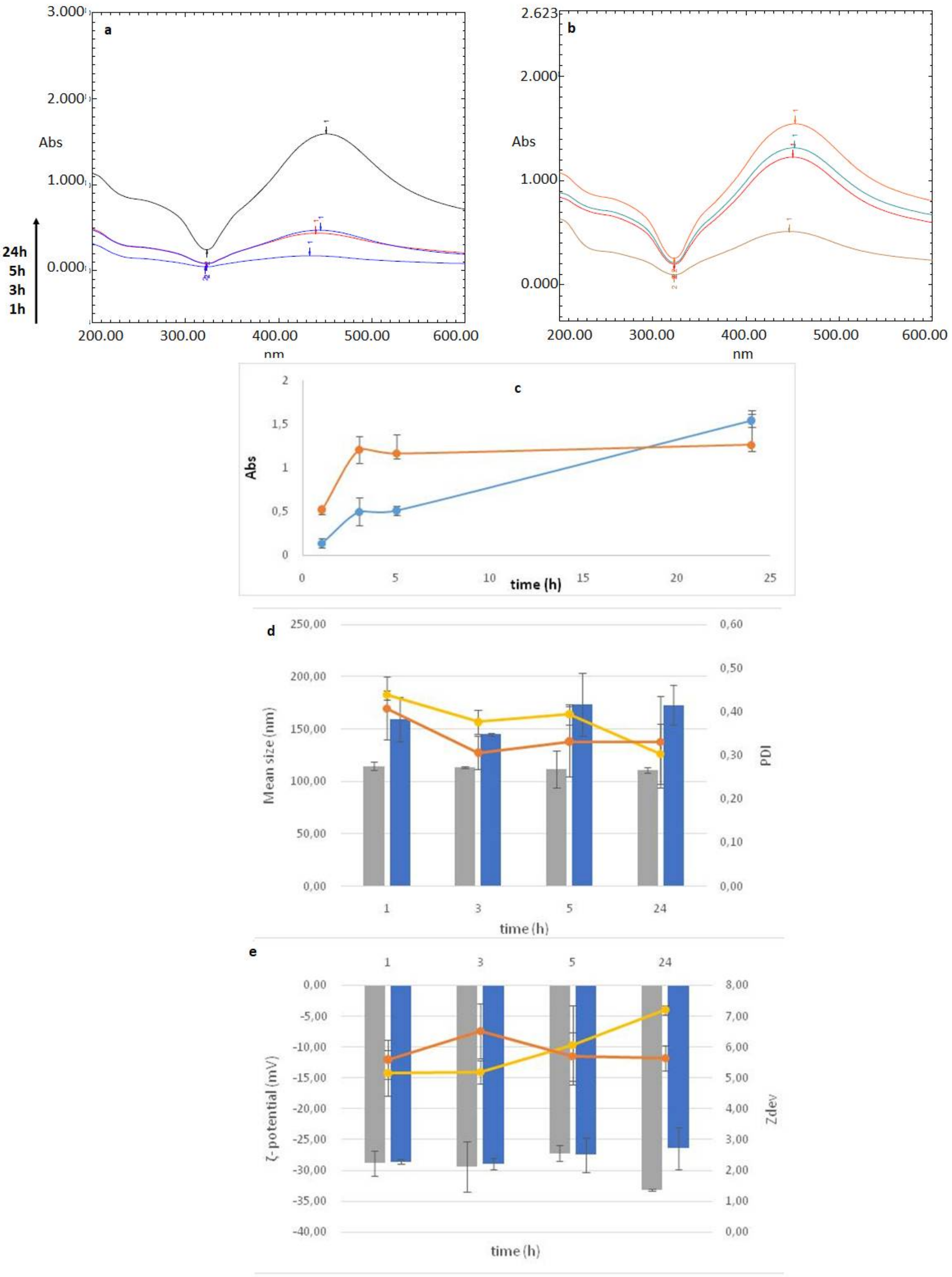

Figure S6. Study of the synthesis of Sage AgNPs monitoring the intensity of the absorbance of the reaction mixture (a) without or (b) with heating, (c) the alteration of absorbance over time, (d) the mean size (column) and PDI (line) and (e) the ζ-potential of the nanoparticles prepared without (■–) or with (■–) heating at predetermined time points.

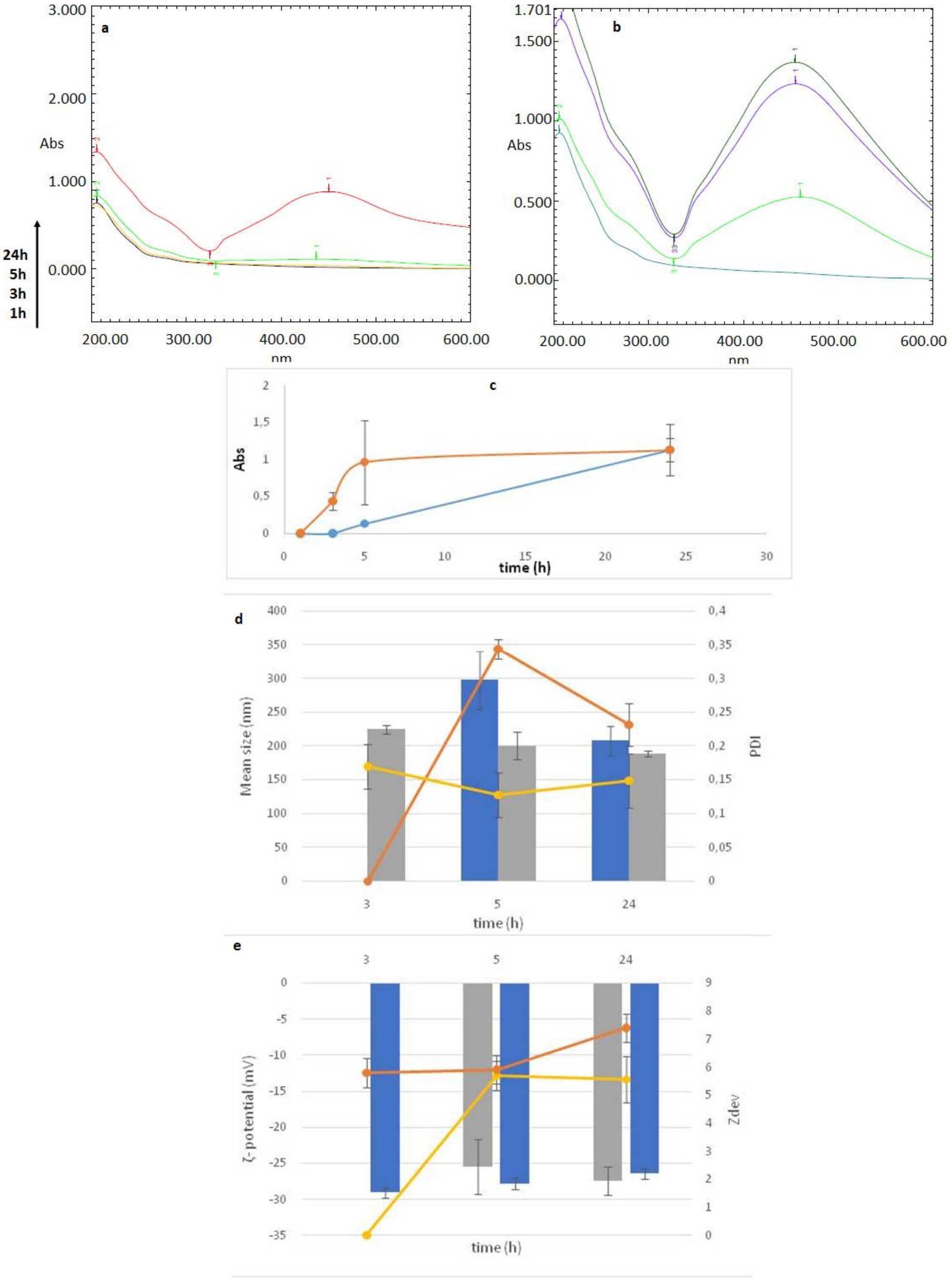

Figure S7. Study of the synthesis of Sea buckthorn AgNPs monitoring the intensity of the absorbance of the reaction mixture (a) without or (b) with heating, (c) the alteration of absorbance over time, (d) the mean size (column) and PDI (line) and (e) the ζ-potential of the nanoparticles prepared without (■–) or with (■–) heating at predetermined time points.

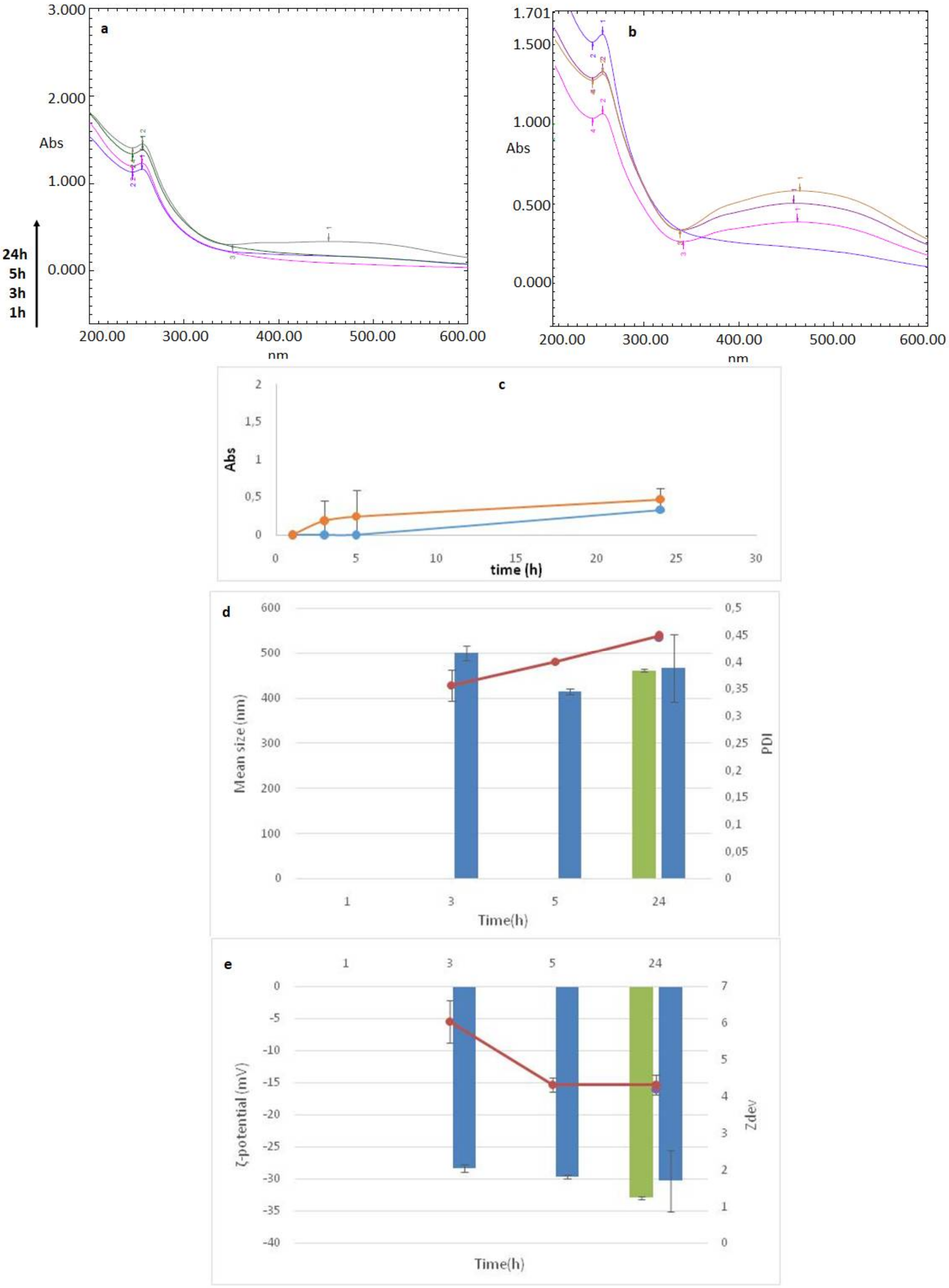

Figure S8. Study of the synthesis of Calendula AgNPs monitoring the intensity of the absorbance of the reaction mixture (a) without or (b) with heating, (c) the alteration of absorbance over time, (d) the mean size (column) and PDI (line) and (e) the ζ-potential of the nanoparticles prepared without (■–) or with (■–) heating at predetermined time points.

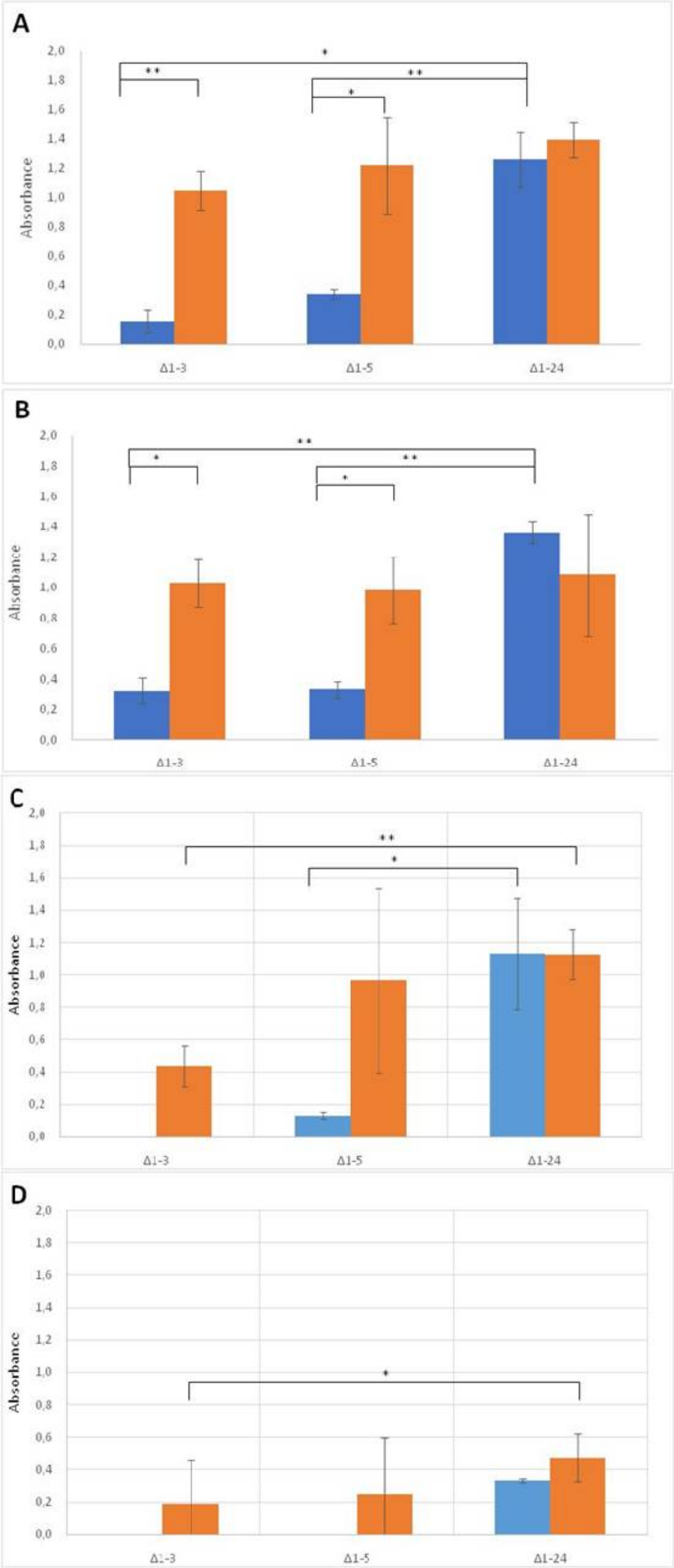
A
B
C
D
Absorbance
2,0
1,8
1,6
1,4
1,2
1,0
0,8
0,6
0,4
0,2
0,0
Δ1-3
Δ1-5
Δ1-24
*
**

Figure S9. Comparison of the alteration of the UV/Vis absorbance of (a) Dittany, (b) Sage, (c) Sea buckthorn and (d) Calendula AgNPs at every time point with (■) or without (■) heating (*: $p < 0.05$ **: $p < 0.005$).

a

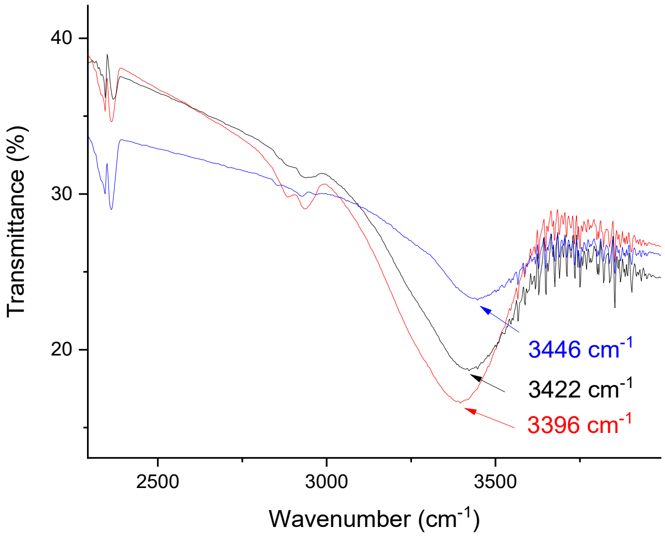

b

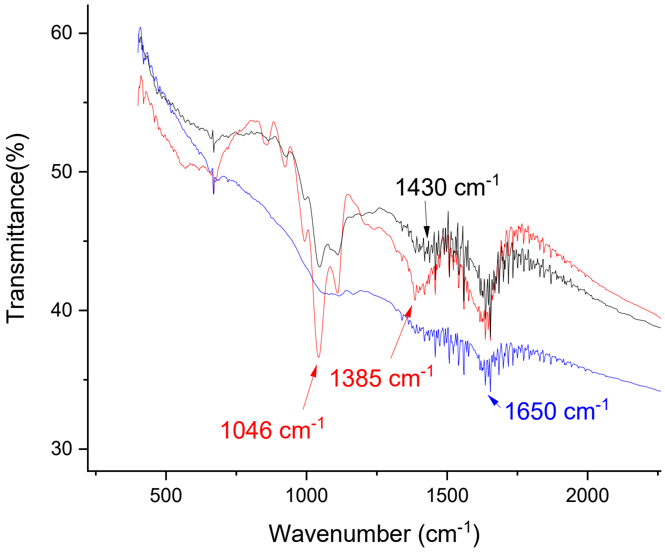

Figure S10. FT-IR spectra of Calendula AgNPs (--), Calendula extract (--) and $NaBH_4$ AgNPS (--), in the spectral area (a) 2500 – 4000 cm^{-1} and (b) 500 – 2000 cm^{-1}. The shift of the main peak maximum is shown.
